# Leaves to Measure Light Intensity

**DOI:** 10.1002/advs.202304420

**Published:** 2024-07-30

**Authors:** Aliénor Lahlou, Ian Coghill, Mhairi L. H. Davidson, Romain Billon, Fredy Barneche, Dusan Lazar, Thomas Le Saux, Ludovic Jullien

**Affiliations:** ^1^ PASTEUR, Département de chimie, École normale supérieure PSL University, Sorbonne Université CNRS Paris 75005 France; ^2^ Sony Computer Science Laboratories Paris 75005 France; ^3^ Institut de biologie de l'École normale supérieure (IBENS), École normale supérieure CNRS, INSERM, Université PSL Paris 75005 France; ^4^ Jardin des Plantes de Paris Museum National d'Histoire Naturelle Paris 75005 France; ^5^ Department of Biophysics, Faculty of Science Palacký University Olomouc 77900 Czech Republic

**Keywords:** actinometry, fluorescence, green materials, irradiance, light intensity, photoactive materials

## Abstract

Quantitative measurement of light intensity is a key step in ensuring the reliability and the reproducibility of scientific results in many fields of physics, biology, and chemistry. The protocols presented so far use various photoactive properties of manufactured materials. Here, leaves are introduced as an easily accessible green material to calibrate light intensity. The measurement protocol consists in monitoring the chlorophyll fluorescence of a leaf while it is exposed to a jump of constant light. The inverse of the characteristic time of the initial chlorophyll fluorescence rise is shown to be proportional to the light intensity received by the leaf over a wide range of wavelengths and intensities. Moreover, the proportionality factor is stable across a wide collection of plant species, which makes the measurement protocol accessible to users without prior calibration. This favorable feature is finally harnessed to calibrate a source of white light from exploiting simple leaves collected from a garden.

## Graphical abstract

Leaves provide an easily accessible green material to robustly calibrate light intensity. Under exposure to a jump of constant light, their ChlF exhibits a fast evolution at a characteristic time, which is inversely proportional to the light intensity received by the leaf over a wide range of wavelengths and intensities. This favorable feature is harnessed to calibrate a source of white light.

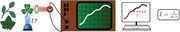

## 1 Introduction

Quantitative measurement of light intensity is presently highly demanded by physicists, biologists, and chemists involved in fields as diverse as production of molecules and materials, design of medical protocols, optical bioimaging, optogenetics, or photocatalysis.^[^ ^1^, ^2^, ^3^, ^4^ ^]^ Indeed this measurement is essential to compare scientific results from different sources and to ensure their reproducibility.

Materials exhibit multiple interactions with light thereby altering the material properties or the incoming electromagnetic field such as (non)linear optics, photorefractivity, photochromism, photovoltaics, photocatalysis, magneto‐optics, etc.^[^ ^5^, ^6^, ^7^, ^8^, ^9^ ^]^ Hence, they provide various designs for measuring light intensity. Selenium and silicon light meters use photovoltaic sensors generating a voltage proportional to light exposure, whereas cadmium sulfide light meters exploit a photoresistor sensor whose electrical resistance changes proportionately to light exposure. As an alternative method, actinometers are photoactive materials, which provide information on light intensity from extracting a time after optically analyzing the time course of the extent of a photochemical reaction with a known quantum yield.^[^ ^10^ ^]^

Light meters and actinometers are powerful tools but they suffer from limitations.^[^ ^11^, ^12^ ^]^ Measuring light intensity with light meters is fast and can be achieved over a wide range of wavelengths and incident light intensities. Yet, this measurement requires a specific instrument and the independent determination on the illumination spot size.^[^ ^13^, ^14^ ^]^ Actinometers often rely on the low sensitive absorbance for reporting and their accessibility is limited for most end‐users.^[^ ^10^, ^15^ ^]^ Here, we are interested in studying a light‐measuring system, which combines the attractive features of light meters and actinometers while addressing some of their limitations.

Plant leaves are abundant green materials. They have been considered to produce silica or other chemicals^[^ ^16^ ^]^ or as a source of components.^[^ ^17^ ^]^ They have also been important actors of bioinspiration (e.g., in relation to specific wettability properties^[^ ^18^, ^19^ ^]^). More generally, photosynthetic organisms have been harnessed to produce living materials.^[^ ^20^ ^]^ However, although strongly photoactive and sensitive to light, they have never been considered for quantitative measurement of light intensity. Here, we show that they act as widely accessible fluorescent actinometers, which are relevant to quantitatively measure light over the whole 400–650 nm visible light window in a broad range of light intensities covering two to three orders of magnitude, without spot size calibration.

## 2 Results

### 2.1 Principle of the Measurement of Light Intensity

In oxygenic photosynthetic organisms, sunlight is collected with an efficient antenna absorbing light in the whole visible wavelength range.^[^ ^21^, ^22^ ^]^ The absorbed energy is conveyed to the photosystem II (PSII) and photosystem I, where it drives a charge separation accompanied by water splitting leading to oxygen evolution, followed by assimilation of carbon dioxide to produce sugars. However, a small part of the absorbed energy (a few percent) is released as fluorescence emission (mostly by chlorophylls, Chl) spanning the 650–800 nm range with an emission maximum around 680 nm and a smaller peak at about 730 nm at room temperature.

When a dark‐adapted leaf is exposed to continuous constant light, the Chl a fluorescence (ChlF) intensity shows characteristic changes.^[^ ^23^ ^]^ It first rises during about a second. Following on from this step, the ChlF level decays over a few minutes as a consequence of several events (changes in redox states of components of the linear electron transport flow, involvement of alternative electron routes, build‐up of a transmembrane pH difference and membrane potential, activation of different nonphotochemical quenching processes, activation of the Calvin–Benson cycle, etc).^[^ ^24^, ^25^, ^26^, ^27^, ^28^ ^]^

Here, we focus on the fast ChlF rise called OJIP rise.^[^ ^29^ ^]^ Mainly coming from PSII,^[^ ^23^, ^29^ ^]^ it primarily reports on the successive reduction of the electron acceptors of the photosynthetic electron transport chain. The ChlF intensity rises in less than 1 s from a minimum level (the O level) to a maximum level (P). Depending on the light level and the experimental conditions, it exhibits one, two, or three intermediate steps identified as local maxima labeled K, J, and I. The first step systematically reflects the light‐limited reaction (reduction of the electron acceptor Q_*a*_). Interestingly, the rate constant of this so‐called photochemical phase has been reported to linearly depend on the intensity of the exciting light^[^ ^30^, ^31^ ^]^ and to not significantly depend on the nature of the photosynthetic organism,^[^ ^32^, ^33^ ^]^ two features that are favorable for exploiting such systems for light measurement. Yet, the reported facts do not presently enable to exploit green leaves for quantitative measurement of light intensity, which is the purpose of the present work.

Considering that the ChlF emission from a leaf is a side‐product of the light‐driven process of charge separation, the rate constant driving the initial step of the ChlF rise is the product of two terms: the cross‐section for PSII photoactivation denoted σ which reflects the capacity to absorb light, and the light intensity/(more precisely irradiance), which is a surfacic power (W m^−2^) alternatively denominated photon flux density (with mol (photon) m^−2^ s^−1^ or E m^−2^ s^−1^; see Experimental Section). Provided that this rate constant *k* – or similarly its characteristic time τ = 1/*k* – can be retrieved from the time evolution of the ChlF rise, *I* can be measured if σ is known since all these parameters are linked in Equation (1).

(1) $$\begin{array}{ccc} I & = & \frac{1}{\sigma \tau } \end{array}$$

Therefore, the goal of this work has first been to measure the value of σ(λ_exc_) over a wide span of excitation wavelengths λ_exc_ covering the near UV‐visible wavelength range. In order to proceed, we first validated an automated protocol for extraction of τ from the initial step of the ChlF rise on a wide variety of green leaves (61 samples). Then, we explored two complementary approaches to extract σ(λ_exc_): i) we first directly measured the values of σ(λ_exc_) at four selected wavelengths spanning the UV–vis wavelength range; ii) we subsequently established an average ChlF excitation spectrum, which has been shown to be relevant for extrapolating the value of σ(λ_exc_). As a final application, we harnessed a leaf for characterizing a light source exhibiting a broad spectrum of light emission.

### 2.2 Protocol for Measuring the Cross‐Section Associated to the Initial Step of the ChlF Rise

In a first step, we established an automated protocol for measuring the cross‐section associated to the initial step of the ChlF rise in leaves by using continuous illumination. The leave samples are prepared according to the protocol described in the Experimental Section. They are first submitted to darkness for 15 min before starting the illumination experiments. This preliminary step empties the photosynthetic electron transport chain and enables to get a reference state of the leaf. After the dark acclimation, the face of the bifacial leaves that is not directly exposed to sunlight (abaxial) is exposed to constant light at wavelength λ_exc_ in the [400 nm; 650 nm] wavelength range, and the ChlF intensity is collected. 680 nm is the maximum of ChlF emission of the photosynthetic apparatus. However, any wavelength between 650 and 750 nm can be used for ChlF reporting, albeit with a lower signal.

ChlF recording is started before turning on illumination (typically 10 ms in our experiments) and the collected ChlF signal *F*(λ_exc_, λ_em_, *t*), is recorded as a function of time over 1 s. The acquisition frequency has to be high enough in order to enable satisfactory sampling of the initial step of the ChlF rise (typically 3 MHz in our experiments). The time evolution of the ChlF signal is then automatically processed (a downloadable application is accessible online with instructions for implementation available at https://github.com/Alienor134/OJIP‐fit and in the Experimental Section). The fitting algorithm exploits the approach of Joly & Carpentier^[^ ^32^ ^]^ to obtain a preliminary estimation of the parameters of the whole ChlF rise. After identifying the onset of the ChlF response (*F*_0_) and the maximum of ChlF (*F*_*M*_), the algorithm iteratively estimates the parameters of the initial step of ChlF rise:

a) In a first step, the algorithm applies an unsupervised fit with Equation (2)
(2) $$\begin{array}{ccc} F(t) & = & F_{0}+A_{1}{\left(1-e^{-t/\tau_{1}}\right)}^{s_{1}}+A_{2}{\left(1-e^{-t/\tau_{2}}\right)}^{s_{2}}\\ & & +\;A_{3}{\left(1-e^{-t/\tau_{3}}\right)}^{s_{3}} \end{array}$$
  in order to retrieve a first estimate τ_1_ of the value of the characteristic time associated to the initial step of the ChlF rise. In this step the ChlF curve is pre‐processed with a smoothing (typically window size 10) and logarithmic subsampling (typically 200 samples per decade);
b) In a second step, it restricts the time window to [0;3τ_1_] and applies the fit given in Equation (3) to the time evolution of the ChlF emission
(3) $$\begin{array}{ccc} F(t) & = & F\left(0\right)+A{\left(1-e^{-t/\tau }\right)}^{s} \end{array}$$
  upon fixing *s* = 1.24^[^ ^32^ ^]^ in order to retrieve a second estimate of the value of the characteristic time τ associated to the initial step of the ChlF rise;
c) In the last step, it restricts the time window to [0;5τ], applies a fitting function given in Equation (4)
(4) $$\begin{array}{ccc} F(t) & = & F\left(0\right)+A{\left(1-e^{-t/\tau }\right)}^{s} \end{array}$$
  to the time evolution of the ChlF emission upon adopting the values of parameters extracted during the second step as starting values, and retrieves the final value of the characteristic time τ associated to the initial step of the ChlF rise.

The light intensity sought for can eventually be extracted by introducing τ and the relevant value of the cross section of photoconversion σ(λ_exc_) in Equation (1).

As a solid criterion allowing end‐users to establish the relevance of their measurement of light intensity, we eventually suggest exploiting the ratio (*F*_m_ − *F*_0_)/*F*_m_, which involves the minimal (*F*_0_) and maximal (*F*_m_) values gathered from the time evolution of the ChlF signal *F* from the illuminated leaf over a second. The ratio reflects the maximum quantum yield of PSII photochemistry^[^ ^34^ ^]^ and a fully developed and healthy plant exhibits a ratio of about 0.75 – 0.84.^[^ ^35^ ^]^ Consequently, only measurements leading to ratios above 0.75 should be retained to reliably extract light intensity by using our reported average value of the cross‐section for photoactivation of the photosynthetic apparatus.

### 2.3 Measurement of the Cross Section Associated to the Initial Step of the ChlF Rise at 470 ± 10 nm

**Figure 1:**
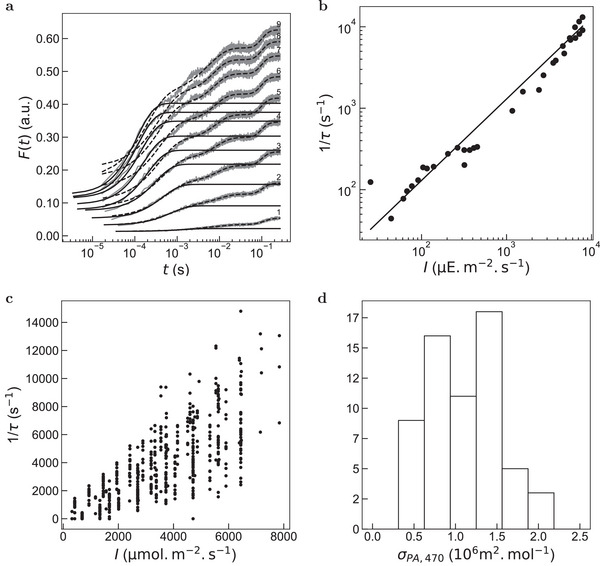
Validation of an automated protocol for extraction of τ from the initial step of the ChlF rise and determination of the cross section associated to the initial step of the ChlF rise from dark acclimated leaves upon constant illumination at 470±10 nm. a) Time evolution of the ChlF emission from a dark acclimated leaf (genus *Chelidonium*) upon illumination at various light intensities (from 1 to 9 in mE m^−2^ s^−1^ (in W m^−2^): 0.3 (75), 1.2 (305), 2.4 (610), 3.5 (890), 4.6 (1200), 5.5 (1400), 6.4 (1600), 7.2 (1800), 7.8 (2000)). A moving average was applied on the experimental data (grey lines) before subsampling with window sizes of 50 and 10 for the fits given in Equation (2) (dashed lines) and (3) (solid lines), respectively; b) Dependence of the inverse of the characteristic time τ associated to the the initial step of the ChlF rise from a dark acclimated leaf (genus *Chelidonium*) retrieved from the monoexponential fit on the light intensity in the range 20–8000 µE m^−2^ s^−1^ (5–2000 W m^−2^). Markers: Experimental data; Solid line: Linear fit. The extracted slope is (1.30 ± 0.05) × 10^6^ m^2^ mol^−1^ where the uncertainty is the standard deviation of the regression coefficient for an ordinary least square regression; c) Dependence of the inverse of the characteristic time τ associated to the initial step of the ChlF rise retrieved from the monoexponential fit on the light intensity for various dark acclimated leaves picked‐up from endemic and imported species grown in different soils, under different illuminations and microclimates over the January 2022 – September 2023 period (61 samples); d) Distribution of the cross‐section associated to the initial step of the ChlF rise from the dark acclimated leaves displayed in (c). The extracted average of the distribution is (1.1 ± 0.8) × 10^6^ m^2^ mol^−1^.

The relevance of the latter measurement protocol and iterative fitting algorithm of the initial step of the ChlF rise has first been evaluated at 470 ± 10 nm on a dark acclimated thin and young leaf from the genus *Chelidonium*, no later than 1 h after its collection. The leaf has been observed under an epifluorescence microscope equipped with a silicon photomultiplier as detector (see Experimental Section). **Figure** **1**a displays representative time evolutions of the normalized fluorescence emission at various intensities of constant light. The fitting protocol was found relevant as displayed in Figure 1a (see also Figure 3a) illustrating different rises of ChlF. As shown in Figure 1b, the inverse of the characteristic time of the photoconversion associated to the initial step of the ChlF rise linearly depends on the light intensity over the whole investigated range. This behavior demonstrates that the process driving the initial step of the ChlF rise evolution is photochemical at least up to 10^−2^ E m^−2^ s^−1^ light intensity. It also enabled us to retrieve the value of (1.30 ± 0.05) × 10^6^ m^2^ mol^−1^ from the slope as a first estimate of the cross‐section of photoconversion at 470 ± 10 nm associated to the initial step of the ChlF rise. With respect to typical orders of magnitude encountered for small photoactive molecules, the later value is extremely high. It is a consequence of the large surface of the antenna over which light is collected and its energy conveyed toward the reaction center of PSII at which charge separation occurs with a very high efficiency.

**Figure 2:**
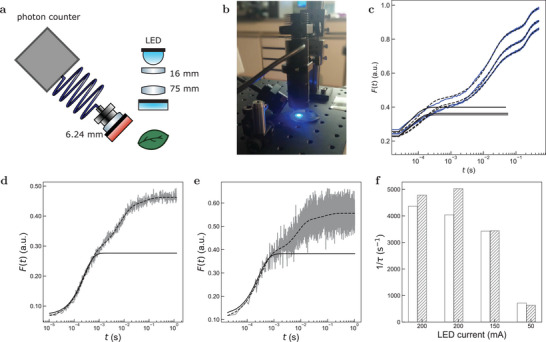
Adapting the protocol for measurement of light intensity with a leaf to alternative optical setups. a–c) Measurement with a simplified set‐up. a,b) Scheme (a) and picture (b) of the simplified set‐up. It consists in an optical fiber equipped with a red fluorescence filter (775/140) connected to a photon detector to access the sample and collect the ChlF. Here the light source to calibrate is simulated by a collimated and filtered LED (470/40 nm); c) Example of ChlF rise obtained with the simplified set‐up. A moving average was performed on the experimental data (blue lines) with window size of 10 followed by a logarithmic subsampling. Applying the fits given in Equation (2) (dashed lines) and (3) (solid lines) respectively yielded τ = (5.1, 5.2, 4.7) × 10^5^ s^−1^. Leaf species: genus *Lonicera*. d‐f) Measurement with an oscilloscope. Fit of the initial step of the ChlF rise performed on the ChlF response acquired with the data acquisition card (d; τ = 209 µs) or the oscilloscope (e; τ = 229 µs). A moving average was performed on the experimental data (grey lines) with window size of 10 (d) or 2 (e) followed by a logarithmic subsampling for the fits given in Equation (2) (dashed lines) and (3) (solid lines) respectively; f) Values of 1/τ retrieved from fitting the time evolution of the ChlF signal collected with the oscilloscope (white) or data acquisition card (striped) for various LED currents (including a repeat at 200 mA). *T* = 293 K.

**Figure 3:**
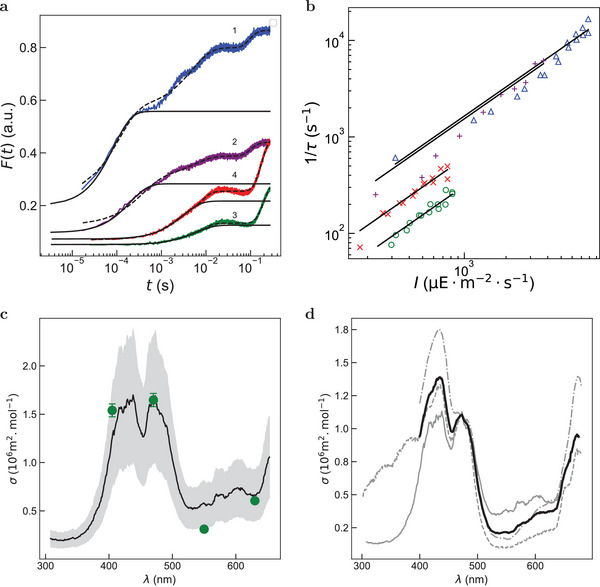
Dependence of the cross‐section associated to the initial step of the ChlF rise from a dark acclimated leaf on the excitation wavelength. Direct determination upon constant illumination at various wavelengths (a,b). a) Time evolution of the ChlF emission from a dark acclimated leaf (genus *Robinia*) upon illumination at various light intensities (from 1 to 4 in mE m^−2^ s^−1^: 7.2, 3.2, 0.73, 0.60) at 470 ± 10 nm (1), 405 ± 7 nm (2), 550 ± 6 nm (3), and 630 ± 9 nm (4). A moving average was performed on the experimental data (grey lines) before subsampling with window sizes of 50 and 10 for the fits given in Equation (2) (dashed lines) and (3) (solid lines) respectively; b) Dependence of the inverse of the characteristic time τ associated to the initial step of the ChlF rise retrieved from the monoexponential fit on the light intensity at 405 ± 7 nm (pluses), 470 ± 10 nm (triangles), 550±6 nm (circles), and 630 ± 9 nm (crosses). Markers: Experimental data; Solid line: Linear fit. The extracted slopes are σ_leaf_(405) = (1.54 ± 0.07) × 10^6^ m^2^ mol^−1^, σ_leaf_(470) = (1.65 ± 0.07) × 10^6^ m^2^ mol^−1^, σ_leaf_(550) = (0.31 ± 0.02) × 10^6^ m^2^ mol^−1^, and σ_leaf_(630) = (0.60 ± 0.02) × 10^6^ m^2^ mol^−1^ where the uncertainties are the standard deviation of the regression coefficient for an ordinary least square regression; Indirect determination from exploiting the fluorescence excitation spectrum of the photosynthetic apparatus (c,d). c) Wavelength dependence of the cross‐section $\sigma (\lambda_{\mathrm{exc}})$ associated to the initial step of the ChlF rise for a *Robinia* leaf as extracted from exploiting its fluorescence excitation spectrum (the grey background reflects a 30% error bar). The graph values are accessible online. Green markers: $\sigma_{\mathrm{leaf}}\left(\lambda_{\mathrm{exc}}\right)$ as measured in (b) (genus *Robinia*). *T* = 293 K; d) Fluorescence excitation spectra of the photosynthetic apparatus from three different photosynthetic organisms. The fluorescence excitation spectra ϵ_norm_ ($\lambda_{\mathrm{em}}=690\pm 2$ nm) have been normalized at 470 nm using the value σ = 1.1 × 10^6^ m^2^ mol^−1^ extracted from Figure 1d. Semi‐dotted grey line: leaf (genus *Robinia*); dotted grey line: *Chlamydomonas reinhardtii (cc124)*; solid grey line: spinach thylakoid.^[^ ^39^ ^]^ The averaged fluorescence spectrum built from the three preceding fluorescence excitation spectra is displayed with a thick black solid line.

Then the iterative fitting algorithm has been successfully applied at different light intensities, on dark acclimated green leaves from 61 samples of plants reflecting a wide variety of photosynthetic species families, of shapes, growth conditions, soils, and microclimates (see Experimental Section). We noticed that the leaves that were not green (*Oxalis triangularis*, *Begonia platanifolia*, purple areas of *Columnea guttata*) were not responding properly to the protocol, probably due to high amount of epidermal anthocyanines. Although anthocyanines absorb mostly green light, they also absorb blue and red light,^[^ ^36^ ^]^ which decreases absorption of chlorophylls. Thus, they were removed from the dataset and we focused on green fully formed leaves. Figure 1c displays the dependence of the inverse of their characteristic time τ associated to the initial step of the ChlF rise and Figure 1d shows the associated distribution of the retrieved cross‐sections. The results enabled us to conclude that using a cross‐section (1.1 ± 0.8) × 10^6^ m^2^ mol^−1^ for measuring light intensity at 470 ± 10 nm gives an accurate value within a factor 2.5 whatever the exploited leaf. Such a low dispersion of the cross‐section was anticipated since the process of light‐induced photochemical reaction in the reaction center of PSII, which is at the core of our protocol for measuring light intensity involves the same molecular structures of the photosynthetic apparatus in all the green plants independently on the climate zone.

To fully confirm the latter derivation, we further studied the distribution of the σ value among leaves of similar size and color collected on three plants from the same species in the same microclimates (see Section S2.1, Supporting Information). We first investigated biological replicates on one‐month old rosettes of *Arabidopsis thaliana* grown under controlled conditions in a laboratory. Interestingly, we could demonstrate that cut leaves (σ = 1.49 ± 0.60 × 10^6^ m^2^ mol^−1^) or leaves on a plant in a pot (σ = 1.42 ± 0.28 × 10^6^ m^2^ mol^−1^) shared similar cross‐sections by respectively using either a microscope or a macroscope allowing to directly run the experiment on the whole plant without pulling‐off its leaves. We additionally retained randomly two plants (genus *Bambusa* and *Chelidonium*) and respectively measured σ = 1.1 ± 0.2 and σ = 1.5 ± 0.2 × 10^6^ m^2^ mol^−1^ by using a microscope to study biological replicates of leaves from different individuals collected in the garden of École Normale Supérieure (45 rue d'Ulm, 75005 Paris) at the same time of the year (March 2024). The standard deviation of σ found for the three examined plants was below the 0.8 × 10^6^ m^2^ mol^−1^ error range provided for the averaged cross‐section reported above, which suggests that the biological diversity of species and microclimates does play a role in the dispersion of σ values. However, this observation also suggests that this dispersion is taken into account in the provided error range, which will allow the user to proceed with randomness for the leaf selection.

To evaluate the robustness of the preceding derivation of the cross‐section associated to the initial step of the ChlF rise at 470 ± 10 nm, we further investigated the variation of the σ parameter between leaves of different ages from two different plant species (see Section 2.2, Supporting Information). While comparing leaves grown over different seasons on the same plants, we concluded that the σ value exhibits some variation on the leaf age but which remains within the error range that we retain and provide for light calibration.

As a final validation step of our method for measuring light intensity with the leaf of a green plant, we challenged our standardized protocol detailed in the Experimental Section by measuring the cross‐section σ associated to the initial step of the ChlF rise at 470 ± 10 nm  on leaves after different time lags between the leaf collection and the measurement (see Section S2.3 Supporting Information). We evidenced that the measurement should be preferentially performed within one hour after the leaf collection.

### 2.4 Minimal Set‐Up for Light Calibration

The preceding series of validating experiments have been performed with an epifluorescence microscope and a fluorescence macroimager. However, such equipments are not necessitated to apply our protocol for measuring light intensity with a leaf.

To support this statement, we first designed an easy‐to‐build‐up setup. Its principal part consists in an optical fiber that can reach the side of the sample to collect the ChlF through a red filter, and a photon detector such as a photon counter or a photodiode (**Figure** **2**a,b), which are classically used to collect the ChlF rise upon illumination.^[^ ^37^, ^38^ ^]^ As displayed in Figure 2c, we could extract the characteristic time τ from analyzing the initial step of the ChlF rise from dark acclimated leaves upon constant illumination at 470 ± 10 nm and show that it provided consistent values of the light intensity: 18 ± 1 mE m^−2^ s^−1^ (4.6 × 10^3^ in W m^−2^) light intensity was retrieved from the τ measurement by using σ = 1.1 × 10^6^ m^2^ mol^−1^ whereas 11 mE m^−2^ s^−1^ was estimated from a calibration relying on a powermeter at the position of the leaf and a ruler to recover the illumination spot size.

On another aspect, the acquisition frequency of the photodetector has to match the dynamics of the initial step of the ChlF rise to yield meaningful information on the light intensity. For blue light in the range 50–10000 µE m^2^ s^−1^ (10–2500 W m^2^), the characteristic time τ varies between 50 ms and 50 µs. During the preceding microscope experiments, we used a data acquisition card to collect the ChlF and subsequently extract the cross‐section for PSII photoactivation from processing its time evolution. Since this equipment might not be available for fast acquisition of the fluorescence signal, we alternatively used an oscilloscope to collect the initial step of the ChlF rise. A *Robinia* leaf was placed under the epifluorescence set‐up and illuminated at 470 nm. We measured the output of the detector collecting the fluorescence signal, which was connected to both an oscilloscope (37 Hz) and the data acquisition card (3 MHz) during the same experiment. As displayed in Figure 2d,e, we observed an accurate match between the outputs for three different values of the current input (Figure 2e), which demonstrates that an oscilloscope can be used to perform the light calibration with a leaf.

Hence, our simple protocol for measuring light intensity with a randomly picked leaf can be implemented with a setup comprising an optical fiber, a red optical filter, a photon detector, and an oscilloscope. Most of these elements can be found in almost every laboratory and do not require extra investments.

### 2.5 Dependence of the Cross‐Section Associated to the Initial Step of the ChlF Rise on the Excitation Wavelength

#### 2.5.1 Direct Measurement

The absorption spectrum of the photosynthetic apparatus is broad, which is favorable to make measurements of light intensity over a wide range of wavelengths. Hence, the dependence of the cross‐section associated to the initial step of the ChlF rise on the excitation wavelength has been investigated with the epifluorescence setup equipped with four different LEDs emitting at 405 ± 7 nm, 470 ± 10 nm, 550 ± 6 nm, and 630 ± 9 nm with collection of emitted fluorescence at 775 ± 70 nm.

**Figure** **3**a displays representative time evolutions of the normalized fluorescence emission at various light intensities of the four LEDs from a dark acclimated leaf (genus *Robinia*) observed with the photodetector under the epifluorescence microscope. These time evolutions have been fitted with the preceding iterative fitting algorithm in order to retrieve the characteristic time τ associated to the initial step of the ChlF rise. Figure 3b displays the dependence of the inverse of τ retrieved from processing the corresponding evolutions. It was again found linear over the whole range of investigated light intensities. We extracted (1.54 ± 0.07) × 10^6^ m^2^ mol^−1^, (1.65 ± 0.07) × 10^6^ m^2^ mol^−1^, (0.31 ± 0.02) × 10^6^ m^2^ mol^−1^, and (0.60 ± 0.02) × 10^6^ m^2^ mol^−1^ for the cross‐sections $\sigma_{\mathrm{leaf}}\left(\lambda_{\mathrm{exc}}\right)$ associated to the purple, blue, green, and red‐orange lights, respectively.

#### 2.5.2 Exploitation of the Fluorescence Excitation Spectrum of the Photosynthetic Apparatus

To further expand the range of wavelengths at which the cross‐sections associated to the initial step of the ChlF rise from a dark acclimated leaf would be available, we used fluorescence excitation spectra.

We first recorded the fluorescence excitation spectrum of the photosynthetic apparatus of a leaf from the genus *Robinia*. As shown in Figure 3c, it exhibits strong values in the purple‐blue and red wavelength ranges and a minimum at green wavelengths. Upon assuming the quantum yield driving the initial step of the ChlF rise to not depend on the excitation wavelength, we then used the fluorescence excitation spectrum of the leaf to compute the wavelength dependence of the cross‐section $\sigma_{\mathrm{leaf},\mathrm{exc}}\left(\lambda_{\mathrm{exc}}\right)$ associated to the initial step of the ChlF rise by fixing the value of the cross‐section to 1.65 × 10^6^ m^2^ mol^−1^ at 470 nm as a reference (value measured for the *Robinia* leaf – Figure 3b). At 405, 550, and 630 nm, we obtained (1.2 ± 0.4) × 10^6^ m^2^ mol^−1^, (0.6 ± 0.2) × 10^6^ m^2^ mol^−1^, and (0.7 ± 0.4) × 10^6^ m^2^ mol^−1^ respectively, which were in line with the measured values $\sigma_{\mathrm{leaf}}\left(\lambda_{\mathrm{exc}}\right)$ reported above (Figure 3c and **Table** **1**). Thus, this reasonable agreement has suggested that the knowledge of the fluorescence excitation spectrum and one σ value at a given wavelength would be sufficient to predict the σ value at any wavelength within the action range (between 400 and 650 nm).

**Table 1:** Photoconversion parameters associated to the initial step of the ChlF rise in a leaf. The normalized excitation coefficient $\varepsilon_{\mathrm{norm}}\left(\lambda_{\mathrm{exc}}\right)$ was extracted from the average fluorescence excitation spectrum displayed in Figure 3d by fixing the value to one at 470 nm. The cross‐sections $\sigma_{\mathrm{leaf}}\left(\lambda_{\mathrm{exc}}\right)$, $\sigma_{\mathrm{leaf},\mathrm{exc}}\left(\lambda_{\mathrm{exc}}\right)$, and $\sigma (\lambda_{\mathrm{exc}})$ have respectively been measured (see Figure 3) and computed at 405, 550, and 630 nm from using respectively the excitation spectra of the leaf and the average excitation spectrum of various photosynthetic organisms displayed in Figure 3c upon fixing the value of the cross‐section of the photoconversion to 1.65 × 10^6^ m^2^ mol^−1^ (for $\sigma_{\mathrm{leaf},\mathrm{exc}}\left(\lambda_{\mathrm{exc}}\right)$) or 1.1 × 10^6^ m^2^ mol^−1^ (for $\sigma (\lambda_{\mathrm{exc}})$) at 470 nm. $I_{\sup}\left(\lambda_{\mathrm{exc}}\right)$ indicates the upper light intensity tested and relevant for a reliable measurement.

$\lambda_{\mathrm{exc}}$	$\varepsilon_{\mathrm{norm}}\left(\lambda_{\mathrm{exc}}\right)$	$\sigma_{\mathrm{leaf}}\left(\lambda_{\mathrm{exc}}\right)$	$\sigma_{\mathrm{leaf},\mathrm{exc}}\left(\lambda_{\mathrm{exc}}\right)$	$\sigma (\lambda_{\mathrm{exc}})$	$I_{\sup}\left(\lambda_{\mathrm{exc}}\right)$
(nm)		(10^6^ m^2^ mol^−1^)	(10^6^ m^2^ mol^−1^)	(10^6^ m^2^ mol^−1^)	(E m^−2^ s^−1^ (W m^2^))
405	1.0 ± 0.2^a)^	1.54 ± 0.07^b)^	1.2 ± 0.6^c)^	1.1 ± 0.8^d)^	10^−2^(3000)^e)^
470	1.0 ± 0.2^a)^	1.65 ± 0.07^b)^	1.65 ± 0.6^c)^	1.1 ± 0.8^d)^	10^−2^(2600)^e)^
550	0.2 ± 0.1^a)^	0.31 ± 0.03^b)^	0.6 ± 0.3^c)^	0.22 ± 0.16^d)^	10^−2^(2200)^e)^
630	0.4 ± 0.1^a)^	0.60 ± 0.04^b)^	0.7 ± 0.4^c)^	0.44 ± 0.32^d)^	10^−2^(1900)^e)^

a) The error on the average fluorescence excitation spectrum $\varepsilon_{\mathrm{norm}}\left(\lambda_{\mathrm{exc}}\right)$ arises from the analysis of the data displayed in Figure 3d;

b) The error on σ_leaf_ is given by the standard deviation of the linear coefficient using an ordinary least squares regression;

c) The error on $\sigma_{\mathrm{leaf},\mathrm{exc}}\left(\lambda_{\mathrm{exc}}\right)$ has been computed by taking into account the propagation of the error on the fluorescence excitation spectrum of the leaf used for the experiment, which has been evaluated to 30% of the value at 470 nm;

d) The error on $\sigma (\lambda_{\mathrm{exc}})$ has been computed by taking into account the distribution of the σ values displayed in 1d and the error on the average spectrum;

e) Extracted from the value of $I_{\sup}\left(\lambda_{\mathrm{exc}}\right)$ by using $I_{\sup}\left(\lambda_{\mathrm{exc}}\right)=I_{\sup}\left(\lambda_{\mathrm{exc},\mathrm{ref}}\right)\times \frac{\varepsilon (\lambda_{\mathrm{exc},\mathrm{ref}})}{\varepsilon (\lambda_{\mathrm{exc}})}$ where $\lambda_{\mathrm{exc},\mathrm{ref}}$ designates the wavelength at which the extraction of the cross‐section of photoconversion has been performed ($\lambda_{\mathrm{exc},\mathrm{ref}}=470$ nm).

To evaluate the variance of the fluorescence excitation spectrum of the photosynthetic apparatus among photosynthetic organisms, we further recorded the fluorescence excitation spectrum from a culture of microalgae (*Chlamydomonas reinhardtii cc124* in exponential growth phase) and superposed the reported fluorescence excitation spectrum from a spinach thylakoid solution.^[^ ^39^ ^]^ As displayed in Figure 3d, the three fluorescence excitation spectra exhibit similar shapes. Hence, we built an average fluorescence excitation spectrum of the photosynthetic apparatus (Figure 3d), which has been subsequently used for extracting estimates of the light intensity over a wide range of wavelengths (accessible online).

Altogether, the preceding agreements have suggested that the average fluorescence excitation spectrum could be exploited to compute an estimate of the wavelength dependence of the cross‐section associated to the initial step of the ChlF rise at any wavelength between 400 and 650 nm from the value measured at 470 nm. Hence, upon using the average value 1.1 × 10^6^ m^2^ mol^−1^ of the cross‐section at 470 nm determined in Figure 1d as a reference, we eventually used the normalized fluorescence excitation spectrum shown in Figure 3d to build an average wavelength dependence of the cross‐section $\sigma (\lambda_{\mathrm{exc}})$ associated to the initial step of the ChlF rise (see Figure 3d). Table 1 sums up the relevant parameters which have to be used for measuring the light intensity at various wavelengths with a leaf.

### 2.6 Application: Characterization of the Spectral Light Intensity of a White Light Source

Manuscripts exploiting photochemistry often report on the power of light sources together with geometrical indications of their position with respect to the samples. However, this information is limited to quantitatively retrieve the incident light intensities, which drives the kinetics of the photochemical reactions. Moreover, even when the latter is provided, its spectral distribution may be lacking. Hence, an attractive application envisioned for a leaf is to measure the incident spectral light intensity of a light source at a sample. More precisely, we addressed the challenging calibration of a white LED by benefiting from the broad light absorption of the photosynthetic apparatus, which covers the whole visible range (Figure 3c).

**Figure 4:**
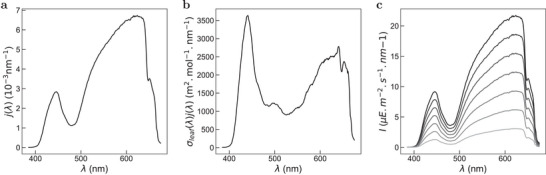
Characterization of the spectral light intensity of a white LED by a leaf‐mediated measurement. a) Normalized emission spectra (*j*(λ) = *S*(λ)/*S*) of the white LED; b) Product of the scaled spectrum σ_leaf_(λ) by the normalized emission spectrum *j*(λ) to generate the action spectrum of the white LED on the photosynthetic apparatus of the leaf, σ_leaf_(λ) × *j*(λ); c) Scaled spectral photon flux of the white LED for current levels 55, 111, 166, 222, 277, 333, 388 mA (light to dark) predicted from $S_{I}={\left(\tau \int_{\lambda_{\min}}^{\lambda_{\max}}\sigma_{\mathrm{leaf}}\left(\lambda \right)j\left(\lambda \right)d\lambda \right)}^{-1}$. The corresponding *S*_*I*_ values are 0.5, 0.9, 1.4, 1.8, 2.3, 2.7, 3.2 mE m^−2^ s^−1^. *T* = 293 K. See Experimental Section.

In a first step, we recorded the unscaled emission spectrum *S*(λ) of the LED (filtered above 665 nm to avoid spectral overlap with the fluorescence emission of the leaf), which has been normalized by its integral to yield the normalized emission spectrum *j*(λ) = *S*(λ)/*S* displayed in **Figure** **4**a. The average σ_leaf_(470 nm) = 1.1 × 10^6^ m^2^ mol^−1^ cross‐section associated to the initial step of the ChlF rise from dark‐acclimated leaves retrieved from Figure 1d was used to scale the average excitation spectrum displayed in Figure 3d and generate the scaled fluorescence excitation spectrum σ_leaf_(λ). The resulting scaled spectrum σ_leaf_(λ) was multiplied by the normalized emission spectrum *j*(λ) to generate the action spectrum of the white LED on the photosynthetic apparatus of the leaf, σ_leaf_(λ) × *j*(λ) (Figure 4b). Then we submitted the leaf to illumination with the white LED at different current inputs and recorded the rises of its fluorescence emission at 775 ± 70 nm as a function of time. Each characteristic time τ associated to the initial step of the ChlF rise was used to retrieve the scaling parameter *S*_*I*_ for the corresponding current input (see Experimental Section). Figure 4c displays the resulting stack of scaled emission spectrum of the white LED.

## 3 Discussion

Leaves are subjected to multiple processes driven by light,^[^ ^24^, ^25^, ^26^, ^27^, ^28^ ^]^ which are prone to produce images.^[^ ^40^, ^41^, ^42^, ^43^ ^]^ These processes span over various time scales. The closer the process is to the driving photon absorption, the faster it is to measure and the more reliable it is for measuring light intensity. First, the processes get slower when they become farther in the metabolism of photosynthesis. Hence, to use slower processes for reporting on light intensity would take longer time and reduce the range of measurable light intensity since the photochemical step has to determine the overall kinetics. Moreover, as the processes become located in the metabolism of photosynthesis, they integrate other factors than the sole input of light to govern their extent, which would affect the robustness of the measurement of light intensity.

**Figure 5:**
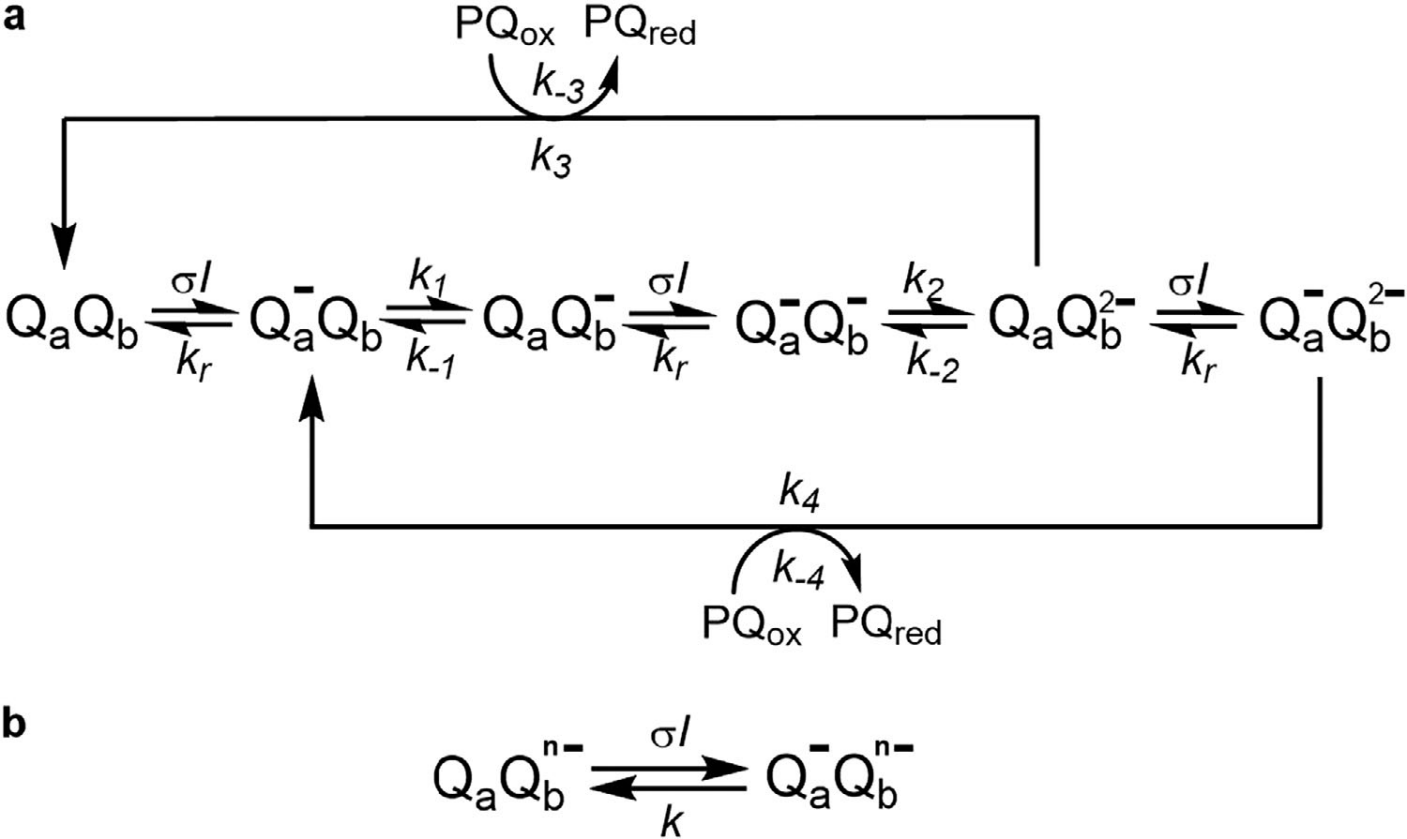
Mechanisms accounting for the time evolution of ChlF. a) Complete kinetic model. Illumination at light intensity *I* drives the reversible reduction of $Q_{a}^{-}$, the reduced first quinone electron acceptor in photosystem II (PSII). In turn, $Q_{a}^{-}$ then reduces Q_*b*_, which is the second quinone electron acceptor in PSII. After accumulation of two electrons at Q_*b*_ and $Q_{b}^{2-}$ protonation by two protons from the stroma, the resulting protonated doubly reduced Q_*b*_H_2_ is released to the plastoquinone pool as PQ_red_. The vacant place in the $Q_{b}^{-}$ pocket of PSII is then filled back by a molecule from the oxidised part of the plastoquinone pool, PQ_ox_, forming thus Q_*b*_ in PSII. Order of magnitude of the rate constants:^[^ ^45^, ^46^ ^]^ σ ≃ 1 × 10^6^ m^2^ mol^−1^, *k*_*r*_ ≃ 1 × 10^4^ s^−1^, *k*_1_ ≃ 5 × 10^3^ s^−1^, *k*_−1_ ≃ 2 × 10^2^ s^−1^, *k*_2_ ≃ 1.5 × 10^3^ s^−1^, *k*_−2_ ≃ 5 × 10^1^ s^−1^, *k*_3_ = *k*_4_ ≃ 1 × 10^2^ s^−1^, *k*_−3_ = *k*_−4_ ≃ 5 × 10^1^ s^−1^; b) Reduced kinetic model. It involves the rate constants σ*I* – where σ designates the cross section associated with the photoactivation – and *k* – which reports on the back reaction (*k*_*r*_) and the relevant electron transfer to Q_*b*_ (*k*_*n*_; *n* = 1 or 2) at the considered light intensity.

When a dark‐adapted leaf is exposed to continuous constant light, the redox reactions of the photosynthetic apparatus are significantly reflected in the initial ChlF rise only for the first second. They are essentially conserved among photosynthetic organisms so as to ensure robustness of the present protocol for measuring light intensity. In particular, the variable ChlF can be modeled by considering that illumination leads to the reversible formation of $Q_{a}^{-}$, the reduced first quinone electron acceptor in PSII, and that it is proportional to the fraction of reduced Q_*a*_.^[^ ^23^ ^]^ At its turn, $Q_{a}^{-}$ then reduces Q_*b*_, which is the second quinone electron acceptor in PSII. After accumulation of two electrons at Q_*b*_ and $Q_{b}^{2-}$ protonation by two protons from the stroma, the resulting protonated doubly reduced Q_*b*_H_2_ is released to the plastoquinone pool as PQ_red_. The vacant place in the $Q_{b}^{-}$ pocket of PSII is then filled back by a molecule from the oxidized part of the plastoquinone pool, PQ_ox_, forming thus Q_*b*_ in PSII. The associated kinetic scheme neglecting protonation of doubly reduced Q*_b_* is displayed in **Figure** **5**a.^[^ ^44^, ^45^ ^]^ Under constant illumination of the initial state Q_*a*_Q_*b*_ (the O level of the ChlF rise) at light intensity *I*, the time evolution of ChlF evidences several kinetic regimes. Upon assuming that ChlF rise only reflects $Q_{a}^{-}$ accumulation, they are associated with the successive accumulations of the states $Q_{a}^{-}Q_{b}$ (J step), $Q_{a}^{-}Q_{b}^{-}$ (I step), and $Q_{a}^{-}Q_{b}^{2-}$ (P step), respectively, which result from the decrease of the rate constants *k*_*i*_ and *k*_−*i*_ when *i* increases. When the light intensity *I* decreases, one progressively observes the disappearance of the fastest steps. The initial rise of ChlF is rate limited by a photochemical activation associated with the rate constant σ*I*. Hence, at the slowest time scale, the time evolution of the ChlF signal can be reliably accounted for with the two‐state kinetic model displayed in Figure 5b.

**Figure 6:**
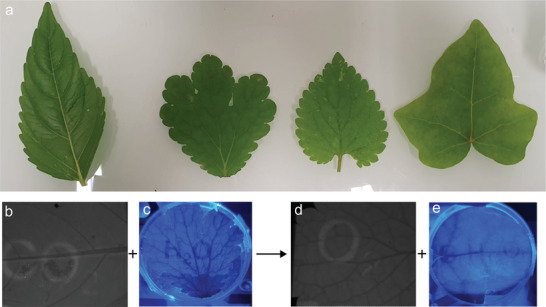
Regulation processes leading to the formation of images. Four leaves picked‐up from the ENS garden (a) have been illuminated for 2 h with a videoprojector, which projected the overall reaction of photosynthesis in black letters against a white background. Then we exposed them to blue light from the back and took a picture of the transmitted light with a cell‐phone camera (c,e), or with collimated blue light and took a picture with a fluorescence filter (b,d). We observe that on the highly‐illuminated areas, the transmission is higher in accordance with chloroplast relocation. The ChlF level is also lower than on the areas not exposed (letters and numbers). This could be due to photoinhibition or non‐photochemical quenching, but also to chloroplast relocation because chloroplasts are responsible for ChlF.

On a longer time scale, other light‐driven phenomena affect the leaf ChlF. In typically 30 s, (non‐) photochemical quenching first decreases ChlF emission.^[^ ^47^ ^]^ However, it is leaf species‐ and physiological state‐specific. Moreover, it cannot be made quantitative without a preliminary calibration. As a result of a longer light exposure, one can observe photoinhibition, which results from the photodegradation of PSII. Hence, the ChlF decreases at the most illuminated zones.^[^ ^48^ ^]^ Interestingly, a linear relationship has been established between the rate‐constant of photoinhibition and light intensity when the leaves are treated with lincomycin, which inhibits the repair of the photodegraded PSII.^[^ ^49^, ^50^, ^51^ ^]^ However, as for non‐photochemical quenching, it is expected to be specific and requesting a demanding calibration. At the 30‐min and longer time scale, blue light further makes chloroplasts to stack on the side of the cells of leaves to minimize their light exposure (avoidance).^[^ ^52^, ^53^ ^]^ As a consequence, a light beam crossing the leaf is less absorbed than when the chloroplasts are equally distributed in the cell. In practice, the three latter phenomena overlap each‐other as illustrated in **Figure** **6**, in which we used a projector to imprint an image of the overall reaction of photosynthesis.^[^ ^43^ ^]^ Among the slowest processes, it is eventually worth to mention starch production. Here light exposure generates contrasted sizes and numbers of starch grains, which has early been used to produce images after extraction of the leaf pigments and iodine staining.^[^ ^40^, ^41^ ^]^ However, the photoproduction and staining of starch is slow (day time scale). Moreover, its robustness has not been investigated and it is not quantitative, which forbids its use for measuring light intensity.

## 4 Conclusion

The experiments reported in this manuscript demonstrate that the fast light‐driven step of ChlF rise occurring at the subsecond time scale in leaves can be robustly harnessed to quantitatively evaluate light intensity. Despite the diversity of leaves, which have been evaluated, similar cross sections associated to the initial step of the ChlF rise have been measured, which illustrates the convergence of the primary process of charge separation in higher plants. Hence, provided that it has been well‐acclimated in darkness, a simple fully‐functional green leaf that has reached maturation randomly chosen in a garden is endowed to yield a good quantitative estimate of light over wide ranges of wavelengths and intensities.

## 5 Experimental Section

### Materials

Several rounds of experiments were performed by selecting leaves of endemic and imported species over two years at different time periods (January, May, July, August and September), grown in different microclimates and with different soil compositions, under different light exposure, at different ages, to average the diversity of the chloroplasts.

**Figure 7:**
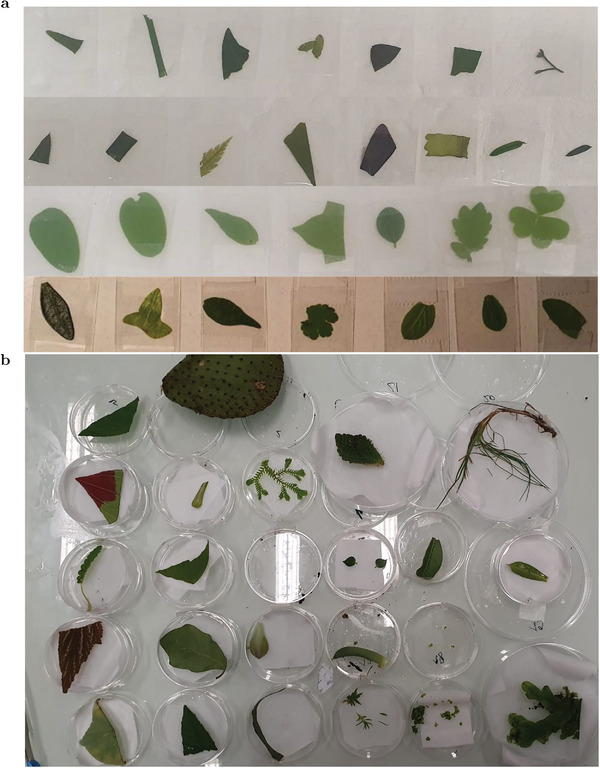
Samples of leaves used to evaluate the distribution of the cross section associated to the initial step of the ChlF rise. a) Leaves from the surroundings of Ecole Normale Supérieure (40 samples in total) used to collect the rise of ChlF. They were placed between two microscope slides taped together. The genus of the leaves shown in the picture were identified with the mobile application PlantNet^[^ ^54^ ^]^: *Trifolium, Chelidonium, Hedera, Robinia, Parietaria, Cotoneaster, Malus, Acer, Liriope, Athyrium, Lonicera, Hesperocyparis, Ginkgo, Plectranthus, Taxus, Platycladus, Oxalis, Olea, Lavandula, Vitis* or family *Poacae*. b) Leaves collected in the different microclimates engineered at the botanic garden of the Museum National d'Histoire Naturelle of Paris (21 samples in total) used to measure the rise of ChlF. They were kept in a humid Petri dish until 20 min before the measurement to allow sample preparation between two glass slides and 15 min dark‐adaptation. The genus of the species collected correspond to *Selaginella, Dictymia, Sinningia, Columnea (green areas), Dischida, Pittosporum, Bocquillonia, Hereroa, Adenium, Monophyllaea, Begonia (later discarded due to its purple color), Graptopetalum, Optunia, Gentiana, Tulipa, Minuartia, Saxifraga, Eremogone, Ramonda, Lemna, Optunia* and *Marchantia*.

Forty leaves were collected in the surroundings of Ecole Normale Supérieure (45 rue d'Ulm, 75005 Paris), both endemic and imported species, under different shadings, identified with the mobile application PlantNet (**Figure** **7**a – genus *Trifolium, Chelidonium, Hedera, Robinia, Parietaria, Cotoneaster, Malus, Acer, Liriope, Athyrium, Lonicera, Hesperocyparis, Ginkgo, Plectranthus, Taxus, Platycladus, Oxalis, Olea, Lavandula, Vitis*, or family *Poacae*). The leaves were collected and analyzed within one hour after their collection. They were cleaned with MilliQ water, dried with absorbing paper, and eventually sandwiched between two 150 µm‐thick glass slides (24 × 60 #1 cover slips – Epredia), which were taped together for the microscope observation. The leaves were always illuminated from below (abaxial – the surface not directly exposed to sunlight on the plants).

To increase the variety of leaves, twenty‐one supplementary samples were collected in the botanic garden of the Museum National d'Histoire Naturelle of Paris (Figure 7b), where imported specimen were grown under different microclimates and growth conditions (controlled humidity levels, light levels, temperature levels, soil composition, and controlled plant competition and watering frequency). These microclimates included arid desert, warm, and humid tropical forest, temperate tropical forest, shadowed pond, alpine climate, with various soil structures including acidic, calcic, rocky, and ultramafic soils. Those microclimates are described in details in the Supporting Information. The genus of the species collected correspond to *Selaginella, Dictymia, Sinningia, Columnea (green areas), Dischida, Pittosporum, Bocquillonia, Hereroa, Adenium, Monophyllaea, Begonia, Graptopetalum, Optunia, Gentiana, Tulipa, Minuartia, Saxifraga, Eremogone, Ramonda, Lemna, Optunia* and *Marchantia*. The *Begonia* was discarded due to its purple color. In this later case, the samples were collected between 9.30 am and 11.30 am and stored in Petri dishes with humid cottons to preserve humidity (tap water). The two leaves collected from the arid microclimate were kept in a Petri dish without humidity. The measurements were performed between 12 am and 7 pm the same day. Each leaf was left in the humid Petri dish in the dark until 20 min before the experiment to allow sample preparation (glass slide preparation presented above) and dark‐adaptation.

The *Arabidopsis thaliana* (Columbia‐0) plants were grown in controlled conditions: 8 h photoperiod (9h00‐17h00), day temperature 21 °C, night temperature: 17–21 °C, light intensity: 100 µE m^−2^ s^−1^ (white LED).

All the samples had been submitted to darkness for 15 min before starting the illumination experiments. Then, a time‐lag of 1 min was left between each measurement at different light intensity on the same leaf.

### Fluorescence Spectrometer

The fluorescence measurements were acquired on a LPS 220 spectrofluorometer (PTI, Monmouth Junction, NJ), equipped with a TLC50 cuvette holder (Quantum Northwest, Liberty Lake, WA) thermoregulated at 25 °C.

The fluorescence excitation spectrum of the leaf was recorded as follows. The leaf was taped on a glass slide and introduced inside a 1 × 1 cm^2^ quartz cuvette at 45° from the incident light beam. The fluorescence emission was collected at 690 nm. The acquired excitation spectrum was corrected from the response of the detector to the glass slide only and it was smoothed with a moving window.

The emission spectra of the LEDs had been recorded by sending the light emitted from the LEDs filtered above 665 nm into the emission pathway of the fluorometer with reduced slits opening.

### Simplified Set‐Up

The epifluorescence microscope, as well as the reference light calibration strategy of the equipment to retrieve the calibrated cross‐section associated with the initial step of the ChlF rise are described in Section S1.1 (Supporting Information). Here, the description of the simplified set‐up that will allow the user to calibrate a light source using the tabulated cross‐sections was provided.

The illumination part consists in a LED (M470L4, Thorlabs, NJ) collimated with a 16 mm lens (ACL25416U‐A, Thorlabs, NJ) and converging with a 75 mm lens (LBF254‐075, Thorlabs, NJ) filtered with a 470/40 nm filter (FF01‐479/40‐25, Semrock, Rochester, NY). The collection part consists in an optical fiber (M29L05, NA = 0.39, 600 μm core diameter, Thorlabs) that harvests the light intensity through a red filter (FF01‐775/140, Semrock) and a 6.24 mm lens (F110SMA‐780, NA = 0.37, Thorlabs, NJ). The output of the fiber was collected by a photon counter (MPPC C13366‐3050GA Hamamatsu, Hamamatsu, Japan) connected to an oscilloscope (RTB2004, Rhode & Schwarz, Munich, Germany) and a data acquisition card (PCI 6374, National Instruments, Austin, TX).

### Protocol for Measuring the Cross‐Section Associated to the *I*nitial Step of the ChlF Rise

The measurement consisted in exposing the conditioned leaves to at least seven different light intensity levels and measuring the ChlF rise over 1 s at 3 MHz. Under blue illumination, it was performed over a range of intensities between 50 µE m^−2^ s^−1^ (13 W m^−2^) and up to 10^−2^ E m^−2^ s^−1^ (2600 W m^−2^) with a time lag of 1 min between each acquisition. Then the initial step of the ChlF rise was fitted with the iterative algorithm to retrieve a relaxation time for each intensity level. The fitness level was evaluated by selecting the ChlF level *F*_0_ at the light onset, and *F*_*M*_ at the ChlF maximum. The $\frac{F_{M}-F_{0}}{F_{M}}$ ratio reflects the maximal quantum yield of PSII photochemistry^[^ ^34^ ^]^ and could be used as a measure of healthness of the leaves. If $\frac{F_{M}-F_{0}}{F_{M}}$ is below 0.75, the datapoint was considered unfit and discarded. The inverse of the relaxation time was fitted against the light intensity with a linear model. To ensure the validity of the fit, a first fit was performed, outlier data points were tested (threshold based on the *z*‐score: two standard deviations). Then a second fit was performed on the remaining data to obtain the final σ value. In practice only one leaf was removed due to an average fitness level of 0.5, and no more than two data points per leaf were removed, either between the first and the second linear fit or due to a low $\frac{F_{M}-F_{0}}{F_{M}}$.

### Implementation of an Application to Retrieve the Light Intensity from the ChlF Kinetics

After acquiring the ChlF kinetics as described in Section 2.2, the user can implement an application we provide online to retrieve the light intensity without needing to manually proceed with the fitting algorithm. In order to execute the application for automated data processing of the time evolution of the ChlF signal from a darkness‐acclimated leaf exposed to constant illumination:

a) Open the address http://127.0.0.1:8050 on a web browser;
b) Drag‐and‐drop the data in .csv format;
c) Select the time and fluorescence columns. Then, the data curve, the fits and the extracted τ value of the initial step of ChlF will be displayed;
d) Select the excitation wavelength used in the experiment, which gives access to the photoactivation cross‐section σ. Then the light intensity sought for *I* was computed using τ and σ, and it appeared on the screen with a factor two error.

### Possible Troubleshootings of the Protocol for Measuring Light Intensity

Three possible troubleshootings for the present protocol for measuring light intensity are listed below:

a) The ChlF rise started decaying after reaching the maximum. The tri‐exponential fit must be stopped at the maximum *F*_m_ for better accuracy;
b) Upon calibrating a red light source, exclude the excitation light from the detected light by using a bandpass filter to collect ChlF that was far enough away from the wavelength range of the emission source (for example using a 700 nm filter to calibrate a 650 nm LED);
c) Check that the observed rise time of the ChlF signal was not driven by the rise time of the light source (e.g., due to heating in LEDs; generally in the µs range^[^ ^55^ ^]^) or of the photodetector by preliminarily analyzing the rise time of the fluorescence signal from a photochemically inert fluorophore upon turning on light.^[^ ^55^ ^]^

### Conversion of Energy Units

In this manuscript, the values of the light intensities in E m^−2^ s^−1^ (or mol of photons m^−2^ s^−1^) were provided. This unit was currently used in actinometry. However, it was not often used in other fields such as optical microscopy, in which the researchers prefer to adopt W m^−2^. Here, the conversion between both units were provided.^[^ ^56^ ^]^ A monochromatic light of wavelength $\lambda_{\mathrm{exc}}$ was considered. Its values in E m^−2^ s^−1^ and W m^−2^ were respectively denoted as $I(\lambda_{\mathrm{exc}},E\;m^{-2}\;s^{-1})$ and $I(\lambda_{\mathrm{exc}},W\;m^{-2})$. The relation between $I(\lambda_{\mathrm{exc}},E\;m^{-2}\;s^{-1})$ and $I(\lambda_{\mathrm{exc}},W\;m^{-2})$ is given in Equation (5)

(5) $$\begin{array}{ccc} I(\lambda_{\mathrm{exc}},W\;m^{-2}) & = & \frac{hcN_{A}}{\lambda_{\mathrm{exc}}}\times I\left(\lambda_{\mathrm{exc}},E\;m^{-2}\;s^{-1}\right)\approx 0.12\times \frac{I(\lambda_{\mathrm{exc}},E\;m^{-2}\;s^{-1})}{\lambda_{\mathrm{exc}}\left(m\right)}\;\;\; \end{array}$$

with the Planck constant *h* = 6.63 × 10^−34^ m^2^ kg s^−1^, speed of light in a vacuum *c* = 3.00 10^8^ m s^−1^, the Avogadro number *N*_*A*_ = 6.02 × 10^23^ mol^−1^, and where $\lambda_{\mathrm{exc}}$ is in m.

### Characterization of the Spectral Light Intensity of a White Light Source

A light source associated with a spectral light intensity *I*(λ) (expressed in E m^−2 ^s^−1^ nm^−1^) spread over [λ_min_;λ_max_] (with the wavelength expressed in nm) was considered. It perpendicularly illuminates a leaf associated to a scaled excitation spectrum leading to its light absorption ϵ(λ) (expressed in m^2^mol^−1^). The rate constant *k* for the initial step of the ChlF rise, as well as the associated characteristic time τ obey Equation (6)

(6) $$k=\frac{1}{\tau }=2.3\int_{\lambda_{\min}}^{\lambda_{\max}}\varepsilon \left(\lambda \right)\phi \left(\lambda \right)I\left(\lambda \right)d\left(\lambda \right)=\int_{\lambda_{\min}}^{\lambda_{\max}}\sigma \left(\lambda \right)I\left(\lambda \right)d\left(\lambda \right)$$

where ϕ(λ) and σ(λ) designate the quantum yield (supposed to be independent on the excitation wavelength) and the cross‐section associated with the  initial step of the ChlF rise.

The normalized emission spectrum *j*(λ) = *I*(λ)/*S*_*I*_ of the light source was introduced, where *S*_*I*_ designates the integral of *I*(λ) over [λ_min_;λ_max_] (Equation (7))

(7) $$\begin{array}{ccc} \int_{\lambda_{\min}}^{\lambda_{\max}}I\left(\lambda \right)d\left(\lambda \right) & = & S_{I} \end{array}$$

and the integral of *j*(λ) over the same wavelength range is equal to one (Equation (8))

(8) $$\begin{array}{ccc} \int_{\lambda_{\min}}^{\lambda_{\max}}j\left(\lambda \right)d\left(\lambda \right) & = & 1 \end{array}$$

Equation (6) yields Equation (9)

(9) $$k=\frac{1}{\tau }=2.3S_{I}\int_{\lambda_{\min}}^{\lambda_{\max}}\varepsilon \left(\lambda \right)\phi \left(\lambda \right)j\left(\lambda \right)d\left(\lambda \right)=S_{I}\int_{\lambda_{\min}}^{\lambda_{\max}}\sigma \left(\lambda \right)j\left(\lambda \right)d\left(\lambda \right)$$

From computing the integral of the action spectrum *AS* of the light source given in Equation (10)

(10) $$AS=\int_{\lambda_{\min}}^{\lambda_{\max}}\sigma \left(\lambda \right)j\left(\lambda \right)d\left(\lambda \right)$$

and measuring the characteristic time τ, one can extract the integral *S*_*I*_ = 1/(τ*AS*) and retrieve the scaled spectral light intensity *I*(λ) = *S*_*I*_ × *j*(λ) sought for.

In practice, one had the dependence of σ(λ) on the wavelength and an unscaled emission spectrum of the light source *S*(λ) (expressed in arbitrary unit) and the goal was to scale it to retrieve the spectral light intensity *I*(λ) (expressed in E m^−2^ s^−1^ nm^−1^) at the sample. The scaling step first involved the normalization of the unscaled emission spectrum *S*(λ) by its integral *S* (Equation (11))

(11) $$\int_{\lambda_{\min}}^{\lambda_{\max}}S\left(\lambda \right)d\left(\lambda \right)=S$$

to yield the normalized emission spectrum *j*(λ) = *S*(λ)/*S*. Then one proceeds as reported above to first compute the integral of the action spectrum *AS* of the light source given in Equation (10), and then retrieve the scaled spectral light intensity *I*(λ) = *S*_*I*_ × *j*(λ) sought for from the measured characteristic time τ and the integral *S*_*I*_ = 1/(τ*AS*).

## Conflict of Interest

The authors declare no conflict of interest.

## Supporting information

Supporting Information

## Data Availability

The data that support the findings of this study are available from the corresponding author upon reasonable request.
