# Supplementary material for: Leaves to Measure Light Intensity
Source: Adv Sci (Weinh). 2024 Jul 30;11(36):2304420. doi: 10.1002/advs.202304420 (PMC11423135; doi:10.1002/advs.202304420)
Supplement: Supplementary file 1 — Supporting Information [file ADVS-11-2304420-s001.pdf]

ADVANCED SCIENCE
Open Access

Supporting Information

for *Adv. Sci.*, DOI 10.1002/advs.202304420

Leaves to Measure Light Intensity

Aliénor Lahlou, Ian Coghill, Mhairi L. H. Davidson, Romain Billon, Fredy Barneche, Dusan Lazar*, Thomas Le Saux* and Ludovic Jullien**

Supplementary materials: Leaves to measure light intensity

Aliénor Lahlou, Ian Coghill, Mhairi L. H. Davidson, Romain Billon, Fredy Barneche, Dusan Lazar,* Thomas Le Saux,* Ludovic Jullien**

Dr. A. Lahlou, Dr. I. Coghill, Dr. T. Le Saux, Prof. Dr. L. Jullien
PASTEUR, Département de chimie, École normale supérieure, PSL University, Sorbonne Université, CNRS, Paris, France
Email Address: Alienor.Lahlou@ens.psl.eu, Thomas.Lesaux@ens.psl.eu, Ludovic.Jullien@ens.psl.eu.

Dr. A. Lahlou
Sony Computer Science Laboratories, Paris, France
Email Address: Alienor.Lahlou@sony.com

Dr. M. L. H. Davidson, Dr. F. Barneche
Institut de biologie de l'École normale supérieure (IBENS), École normale supérieure, CNRS, INSERM, Université PSL, Paris, France

R. Billon
Jardin des Plantes de Paris, Museum National d'Histoire Naturelle, Paris, France

Prof. Dr. D. Lazar
Department of Biophysics, Faculty of Science, Palacký University, Olomouc, Czech Republic Email Address: dusan.lazar@upol.cz

Keywords: *Photoactive materials, Green materials, Light intensity, Irradiance, Actinometry, Fluorescence*

Contents

1 Complement to the Experimental Section **3**

1.1 Epifluorescence microscope 3

1.1.1 System setup 3

1.1.2 Calibration of light intensity 4

1.2 Fluorescence macroscope 4

1.2.1 System Setup 5

1.2.2 Intensity Calibration 6

1.2.3 Measurement Protocol 6

2 Robustness of the cross section associated to the initial step of the ChlF rise at 470±10 nm 7

2.1 Biological replicates on two different optical setups 7

2.2 Impact of the leaf development stage 8

2.3 Impact of the time lag between the leaf collection and the measurement 10

2.4 Microclimates of the botanical garden of the Museum National d'Histoire Naturelle . 10

1 Complement to the Experimental Section

1.1 Epifluorescence microscope

1.1.1 System setup

The measurements of the cross sections associated to the initial step of the ChlF rise from a dark acclimated leaf and of the incident spectral light intensity of a white LED have been performed on a home-built inverted epifluorescence microscope (Figure S1).

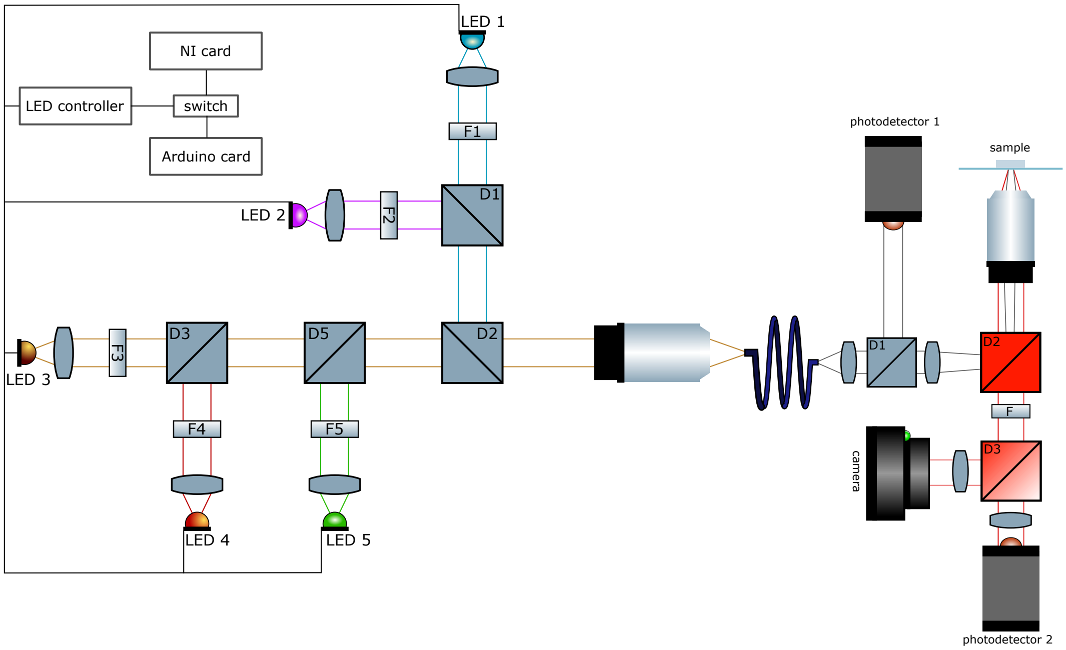

Figure S1: Schematic representation of the epifluorescence microscope.

To illuminate the samples, the lights from high power LEDs (LXZ1-PB01, LHUV-405, LXZ1-PX01, LXM3-PD01; Lumileds, NL) are collimated by high-NA condensers (ACL25416U-A, f = 16 mm, Thorlabs, NJ) and filtered by band pass filters (ET405/20x, ET550/15x; Chroma Technology, VT and FF01-479/40-25, FF01-650/13, Semrock, US) to avoid spectral overlaps. The white LED (LXML-PWN1-0080, Lumileds, NL) was filtered with a short-pass 694 filter (FF01-694/SP, Semrock, Rochester, NY) to eliminate the LED signal that could overlap with the fluorescence of the photosynthetic apparatus.

The quasi-parallel beams are combined with dichroic mirrors (T425LPXR, T505LPXR Chroma Technology, VT and FF01 560 Di01, Semrock, NY) and injected in a 400 μm-core optical fiber using a 20× objective (Nikon, NA = 0.75) to further homogenize the light beams. Light at the output of the fiber is collimated with a 10× objective (Olympus, NA = 0.5) and focused with f = 150 mm lens (AC254-150-A Thorlabs, NJ) at the back focal plane of a 10× imaging objective (Zeiss, NA = 0.5) after being reflected by a dichroic mirror (Di-FF506, Semrock, US). With the white

LED at different current inputs, the rise of the fluorescence emission has been recorded with a 775±70 nm band-pass filter (775/140 single band-pass, Semrock, US).

The LEDs are powered by an LED driver (DC4104, Thorlabs, NJ) controlled by an Arduino Uno card or a DAQ card (PCI 6374, National Instruments, US) depending on the applications. The DAQ card also collects the output signals from detectors. The fluorescence signal has been collected by a photodetector (MPPC C13366-3050GA Hamamatsu, JP). Another photodetector (MPPC C13366-3050GA) is used to collect part of the light excitation that reaches the sample. For the experiment exploiting the measurement with the oscilloscope, we connected the output of the MPPC measuring ChlF flurorescence to both the oscilloscope (RTB2004, Rohde and Schwarz, DE) and the DAQ card (PCI 6374, National Instruments, US) and measured the output on both detectors during the same experiment (see Figure 2 in the Main Text). The acquisition rates of the DAQ card and the oscilloscope are 3 and 8 MHz respectively. However, after the data export, it results in a 37 kHz sampling rate.

1.1.2 Calibration of light intensity

Two protocols have been used to calibrate the coloured LEDs of the optical setup at its focal plane:

- The purple and blue LEDs respectively emitting at 405±7 nm and 470±10 nm have been calibrated with a solution of Dronpa-2 actinometer.[1] They have been used in the range 250–4000 μE.m^{-2}.s^{-1} (80–1200 W.m^{-2}) and 50–8000 μE.m^{-2}.s^{-1} (250–2000 W.m^{-2}) respectively;
- The green and red-orange LEDs respectively emitting at 550±6 nm and 630±9 nm have been calibrated with a powermeter (PM100A, Thorlabs, NJ). Its probe (*S170C* Thorlabs, NJ) was placed at the position of the sample and the excitation wavelength was selected. The collected output is given in Watt. To estimate the surfacic power (W/m^2), we further recorded a scaled image of the illumination spot at the sample position to measure the spot surface (0.25 mm^2 in our configuration where the illumination was narrowed by a diaphragm). The green and red-orange LEDs have been used in the range 250–900 μE.m^{-2}.s^{-1} (50–200 W.m^{-2}) and 200–800 μE.m^{-2}.s^{-1} (40–150 W.m^{-2}).

The rise-time of the LEDs is always faster than a few μs[2] while the dynamics of the initial step of the ChlF rise is in the tenths of microseconds range. Therefore the rise time of the LEDs does not interfere with the recording of the fluorescence rise dynamics.

1.2 Fluorescence macroscope

As a further validation of the light intensity measurement technique introduced in this manuscript, it was applied to attached leaves of *Arabidopsis thaliana* (Columbia-0) plants using a different mea-

surement system: a fluorescence macroscope, specifically built for making measurements on small plants. The measurement was performed on 3 leaves of 4 plants, giving a total of 12 leaves. An image showing one of the plants in place within the system is provided in Figure S2.

1.2.1 System Setup

The arrangement of the optical components of the system is shown in Figure S2, in the form of a computer rendered CAD model, created in Rhinoceros 3D (Robert McNeel & Associates, Seattle, WA, US). The setup consists of separated illumination and sensing paths. The sensing path consists of a macroscope objective (1X/WF, Nikon, Tokyo, Japan), to collimate the light originating from the sample plane; an emission filter (690 nm CWL, 50 nm FWHM; AT690/50m, Chroma Technology Corp., Bellows Falls, VT, US), to pass only the chlorophyll fluorescence; and a camera objective (AF Nikkor 50mm f/1.8D, Nikon, Tokyo, Japan) followed by a condenser lens (ACL2520U-A, Thorlabs Inc., Newton, NJ, US), to focus the collimated light onto the sensor of a silicon photomultiplier module (SiPM, PE3315-WL-TIA-SP, KETEK GmbH, Munich, Germany).

The illumination path consists of a condenser lens (ACL25416U, Thorlabs Inc., Newton, NJ, US) to collect the light from a blue LED (L1RX-BLU1000000000, Lumileds, San Jose, CA, USA) and collimate it for passing through an excitation filter (ET470/40×, Chroma Technology Corp., Bellows Falls, VT, US). Following the filter, the collimated light is injected into the largest entrance of a lightpipe (63-103, Edmund Optics Inc., Barrington, NJ, US) using a plano convex lens (LA1422-A, Thorlabs Inc., Newton, NJ, US). The lightpipe is used to homogenize the input light, providing homogeneous light at the pipe's exit. The exit end of the lightpipe is conjugated to the sample plane through the use of a matched achromatic doublet pair (MAP1040100-A, Thorlabs Inc., Newton, NJ, US). For this work, in order to be able to target single leaves of the small plants, a 1 mm aperture was placed at the exit of the light pipe, such that the illumination took the form of a 2.5 mm spot at the sample plane. Images, or a description, of the optomechanical components used to hold the optical components in place are not described here. It should however be noted that the system features a black box for the sample, which when closed, allows the sample to remain in complete darkness during the measurement protocol. In relation to electronics, the LED was connected to an LED driver (DC4104, Thorlabs Inc., Newton, NJ, US) to power it. The current level delivered to the LED, and therefore the intensity level, was controlled using the external modulation feature of the driver. In this mode, the current delivered to the LED depends upon the voltage applied to the driver's external modulation input. The modulation signal was provided using a multifunction data acquisition card (DAQ, USB-1604HS-2AO, Digilent, Pullman, WA, US). The same card was used to read the voltage signal of the SiPM module. The reading of data from the SiPM and writing of AO data for triggering the light pulse was carried out simultaneously, by triggering both the ADC and DAC with the same timer pulse. The SiPM module was supplied with supply and bias voltages (5 V and 0.4 V, respectively) using a power supply (ELC ALR3206D,

Premier Farnell UK Limited, Leeds, UK).

1.2.2 Intensity Calibration

It was targeted to have 7 different light intensity levels (600, 1000, 2000, 3000, 4000, 5000, 6000, and 7000 μE.m^{-2} .s^{-1}). The voltage levels needed to be supplied to the LED driver were determined by varying it until, through a measurement with a power meter (S170C and PM100A, Thorlabs Inc., Newton, NJ, US), the intensity levels were achieved. Following this, the intensity levels achieved with those voltages which were determined in the previous step were adjusted after measurement using a fluorescence based actinometer (a solution of Dronpa-2). [1]

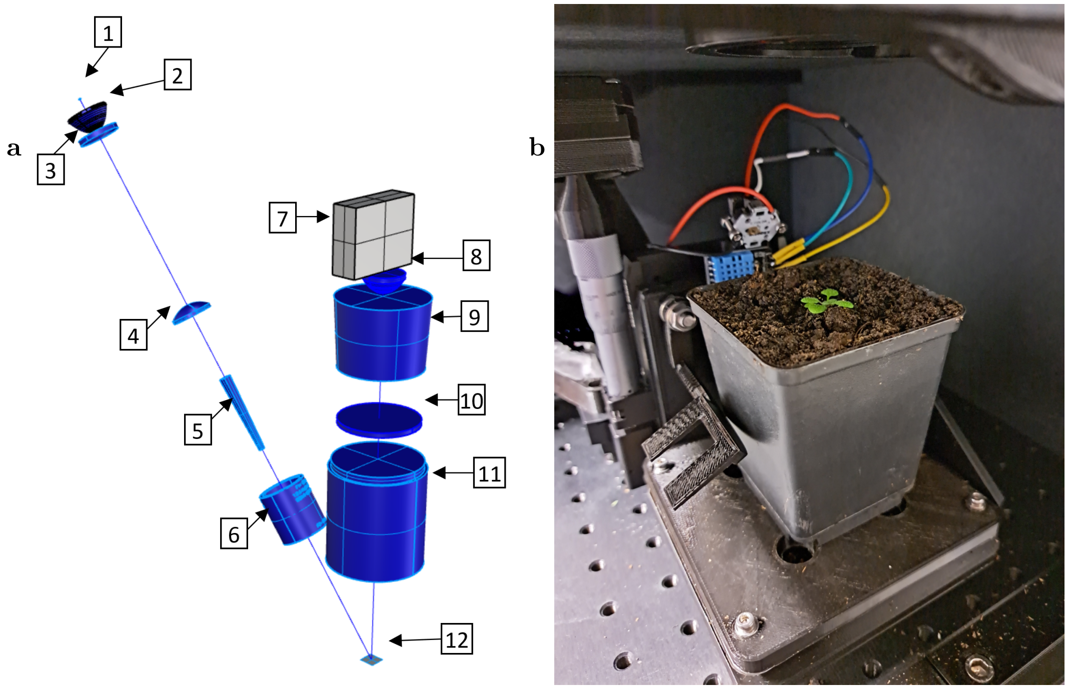

Figure S2: *Macroscope setup.* **a**: CAD rendered diagram of the optical elements composing the macroscope [LEFT] and image of one Arabidopsis plant in place in the system [RIGHT]; 1 – LED, 2 – Condenser, 3 – Excitation Filter, 4 – Condenser, 5 – Lightpipe, 6 – Matched Achromatic Doublet Pair, 7 – Silicon Photomultiplier Module, 8 – Condenser, 9 – Camera Objective, 10 – Emission Filter, 11 – Macroscope Objective, 12 – Sample; **b**: Plant pot under the microscope for direct observation on the leaf of a one-month old *Arabidopsis thaliana* plant

.

1.2.3 Measurement Protocol

After dark adaption, the plants were, one at a time, placed in the macroscope system, and positioned such that the 2.5 mm illumination spot (set at the lowest light intensity possible with the LED driver) was illuminating only a single leaf. Following this, the measurement of OJIP at the first light intensity was started. The measurement consisted of 1 s in the dark, followed by a step function change in light intensity, from darkness to the first light level to be tested, for 1.3 s. As

such, the data contains 1 s where the plant is in the dark, followed by 1.3 s where the plant is exposed to light. After this, the plant was kept in the dark for 60 s, then measurement was repeated at the next light intensity. This was repeated until all light intensities were recorded. The sample rate of the DAQ card was set to 1 MS/s. It should be noted that the results for before and including plant 2 leaf 2 were recorded at 0.5 MS/s, with a light pulse time of 2.6 seconds, and 2 seconds of dark time prior to the pulse. The precise timing of when the LED switches on was seen to vary depending on the light level chosen and, therefore, it was determined in a separate experiment with simply a reflecting surface, the timepoint where the LED switched on, for each light level, and sample rate. This timepoint, plus 50 µs of margin, was used as the beginning of the OJIP curve.

2 Robustness of the cross section associated to the initial step of the ChlF rise at 470±10 nm

2.1 Biological replicates on two different optical setups

To evaluate the robustness of the cross section associated to the initial step of the ChlF rise at 470±10 nm, we first investigated the variation of the σ parameter between leaves collected on different plants of the same species grown in similar conditions at the same time of the year (March 2024).[1] Two species of plants (genus *Bambusa* and *Chelidonium*) were collected in the garden of ENS, while the *Arabidopsis thaliana* plants were grown in laboratory conditions and harvested after one month.

To further evaluate robustness with respect to instrumental changes, we implemented the measurement protocol on two different instruments, the microscope (see subsection 1.1) and the macroscope (see subsection 1.2). The measurements were sequentially performed: a measurement on a leaf was directly performed on the whole plant in a pot with the macroscope, then the illuminated leaf was pulled-off and measured under the microscope.

The results with the experimental data points are displayed in Figure S3. We observe that the dispersion of the σ value among biological replicates is narrower than for the observations over several species, which suggests that biological diversity over different species does play a role in the dispersion of σ values. The error range provided in the Main Text takes account this diversity, which lowers the precision of the estimation of the light intensity but allows to be robust to randomness when selecting the leaves, in terms of species and microclimates.

[1] Although it was not possible to age them precisely, we chose leaves of similar size and color to identify them as biological replicates.

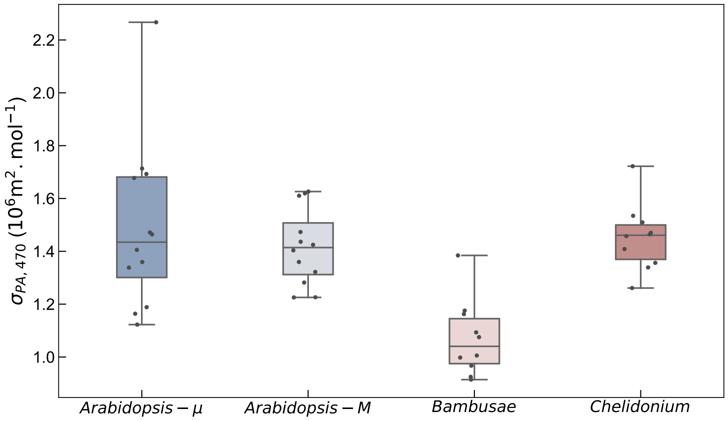

Figure S3: *Value of the cross section σ associated to the initial step of the ChlF rise at 470±10 nm for biological replicates on two different optical setups.* $\sigma = 1.49 \pm 0.60 \times 10^6$ and $\sigma = 1.42 \pm 0.28 \times 10^6$ m^2.mol^{-1} were measured on three leaves collected on four different plant of genus *Arabidopsis* grown in controlled conditions (8 hour photoperiod 9h00–17h00, day temperature 21^oC, night temperature: 17-21^oC, light intensity: 100 μE.m^{-2}.s^{-1} from white LED) with the microscope (μ) and the macroscope (M) respectively. $\sigma = 1.1 \pm 0.2$ and $\sigma = 1.5 \pm 0.2 \times 10^6$ m^2.mol^{-1} were respectively measured with the microscope on leaves of genus *Bambusa* and *Chelidonium* from plants grown in similar conditions collected in March 2024 in the garden of Ecole Normale Supérieure (45 rue d'Ulm, 75005 Paris) (we could not age precisely the leaves; they were selected to have the same size and color).

2.2 Impact of the leaf development stage

We first collected and analyzed similarly conditioned ivy leaves (genus *Hedera*) of different ages (Figure S4a,b). We derived σ values ranging between 0.72 and 1.40×10^6 m^2.mol^{-1} without any significant age-related trend (Figure S4c) from analyzing the light intensity-dependence of the inverse of τ retrieved from the fluorescence kinetics in response to increasing light intensity levels (Figure S4d). To further investigate the impact of the leaf age, we performed a second series of experiments on similarly conditioned leaves from genus *Rosacae* (Figure S4e) and compared the σ value found for leaves grown between February and March 2024 (early Spring) and before the Winter of 2023 or older (Figure S4f). We found $\sigma = 0.96 \pm 0.20 \times 10^6$ m^2.mol^{-1} and $\sigma = 1.25 \pm 0.46 \times 10^6$ m^2.mol^{-1} for the young and old leaves respectively (Figure S4g) from analyzing the light intensity-dependence of the inverse of τ retrieved from the fluorescence kinetics in response to increasing light intensity levels (Figure S4h). The difference of the average values of σ is below the error range provided in the Main Text. Hence, we concluded from both series of experiments that the leaf age does not significantly impact the σ value beyond the expected error range.

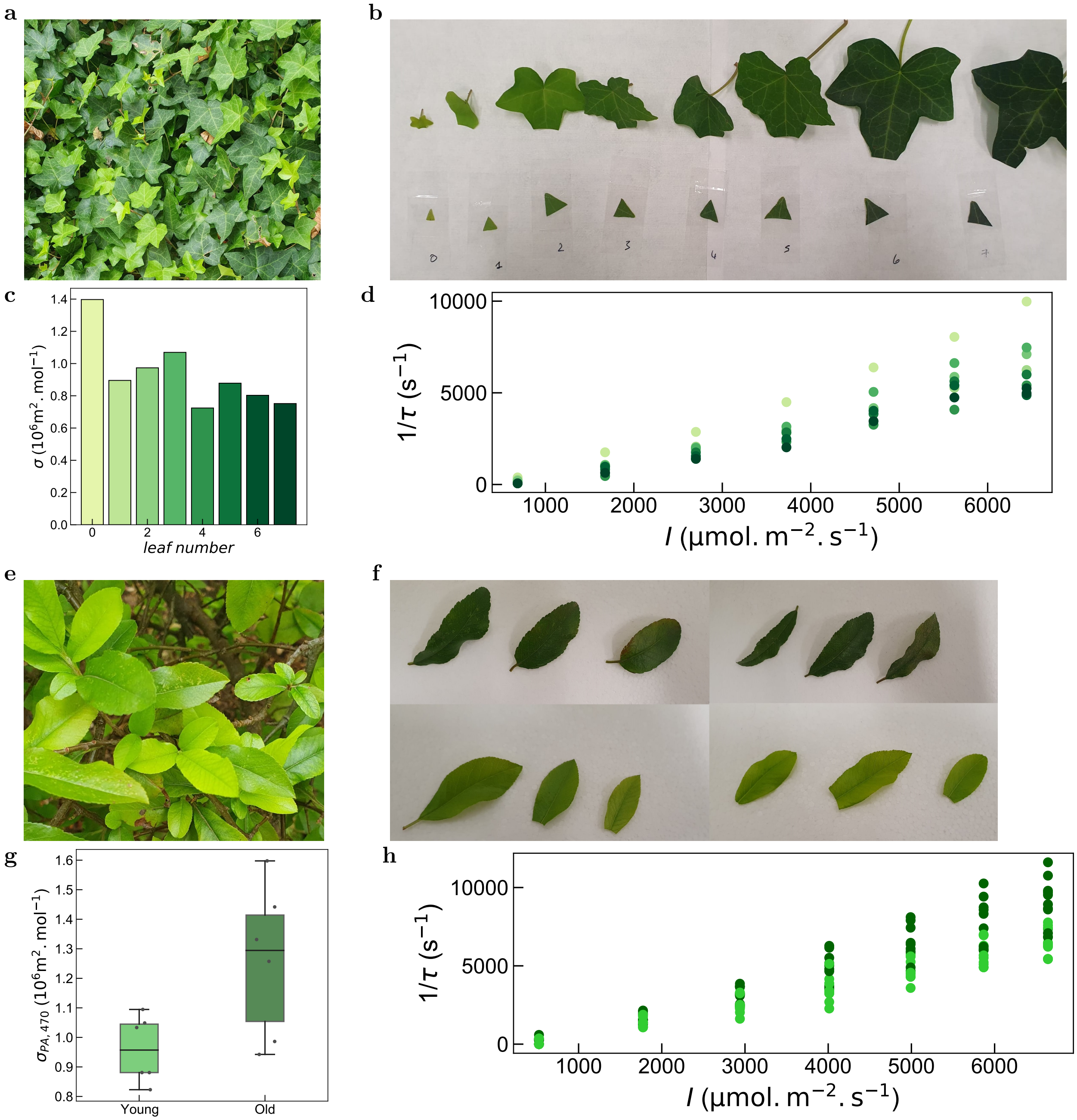

Figure S4: *Evaluation of the impact of the leaf age on the cross section associated to the initial step of the ChlF rise at 470±10 nm.* **a**–**d**: *Genus Hedera.* **a**: Parcel from which the ivy leaves were collected over 1 m^2 at the surface, therefore exposed to the same light intensity. Each leaf was collected and analyzed less than 20 min after being collected to allow sample preparation and 15 min dark-adaptation. **b**: Leaf ordering based on color and size (respectively light-green to dark-green and small to large, to ensure a gradual representation of increased growth time) to correspond to increasing age for increasing leaf number. The apex of each leaf was cut-off with a box cutter and used for the analysis. **c**,**d**: Derivation of the σ value for each collected leaf. The average $\langle\sigma\rangle = 0.94 \pm 0.20\times10^6$ $m^2.mol^{-1}$ has been retrieved from the σ values of all the examined leaves (**c**), which have been extracted from analyzing the dependence of $1/\tau$ on the light intensity for each leaf (**d**; coded by the color in **c**); **e**–**h**: *Genus Rosacae.* **e**: Plant from which the leaves were collected (genus *Rosacae*). Each leaf was collected and analyzed less than 1 h after being collected. **f**: Top: six samples of leaves grown before winter 2023 (referred to as "old"); Bottom: six samples of leaves grown in 2024 (referred to as "young"). The apex of each leaf was cut-off with a box cutter and used for the analysis. **g**,**h**: Derivation of the σ value for each collected leaf. The averages $\langle\sigma\rangle = 0.96 \pm 0.20\times10^6$ $m^2.mol^{-1}$ (young leaves) and $\langle\sigma\rangle = 1.25 \pm 0.46\times10^6$ $m^2.mol^{-1}$ (old leaves) have been retrieved from the σ values of all the examined leaves (**g**), which have been extracted from analyzing the dependence of $1/\tau$ on the light intensity for each leaf (**h**).

2.3 Impact of the time lag between the leaf collection and the measurement

We eventually performed two tests to evaluate whether the time lag between the leaf collection and the measurement impacts the σ value. The first was to collect a leaf, prepare the sample and perform the measurement loop to acquire σ once every hour for seven hours. Figure S5a shows that the value of σ of five distinct leaves of different species does not display any significant variation that could be attributed to the time lag. The second test was performed on twenty one leaves, which were kept in a humid Petri dish before preparing the sample and performing the measurement loop to acquire σ. Figure S5b evidences a slow decay of the σ value on the time lag between the leaf collection and the sample preparation and the measurement.

From the whole series of experiments, we concluded that it is most appropriate to prepare the sample and take the measurement in the hour following the leaf collection.

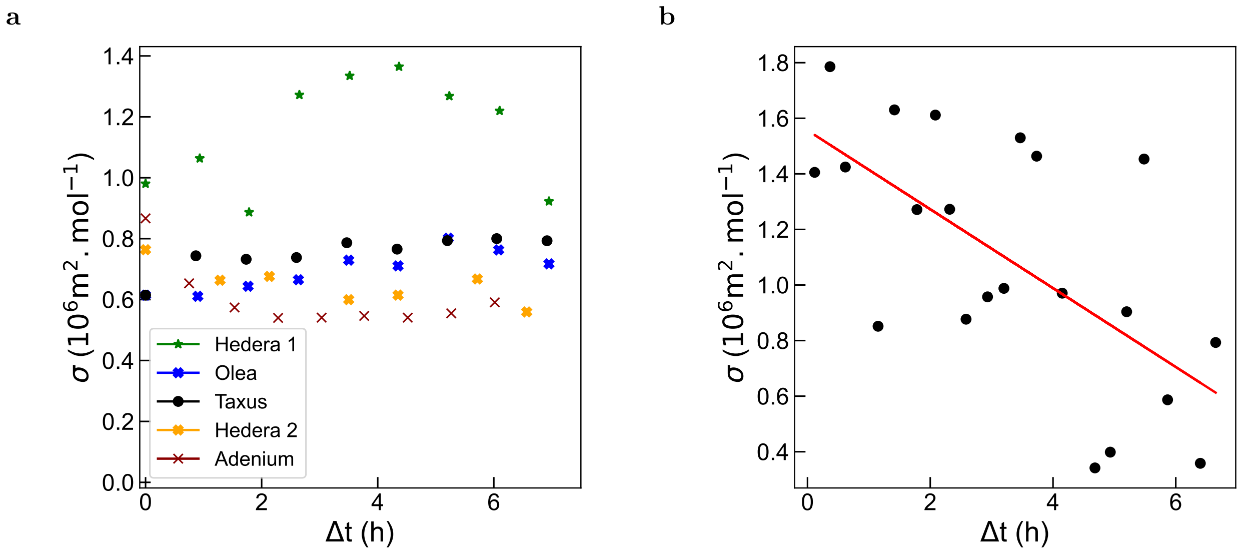

Figure S5: *Evaluation of the impact of the time lag between the leaf collection and the measurement of the cross section associated to the initial step of the ChlF rise at 470±10 nm.* **a**: Evolution of the σ value on the time lag Δt for five samples prepared less than one hour after the leaf collection (from the genus *Hedera* – two different samples, *Olea, Taxus, Adenium*) and exposed to the measurement protocol once every hour for seven hours; **b**: Evolution of the σ value for the twenty-one leaves collected in the botanical garden of the Museum National d'Histoire Naturelle (see Figure 7 in the Main Text) and kept in humid Petri dishes in the dark until 20 min before the measurement to allow sample preparation and 15 min dark-adaptation. The linear fit yields $\sigma = 1.41 - 0.11\Delta t$ where σ and Δt are expressed in 10^6 $m^2.mol^{-1}$ and hour respectively.

2.4 Microclimates of the botanical garden of the Museum National d'Histoire Naturelle

Among the leaves collected, several samples have been collected in the botanical garden of the Museum National d'Histoire Naturelle (see Figure 7b) where the gardeners have engineered multiple microclimates. It allowed us to benefit from a wide diversity of species grown in different condi-

tions that could not have been accessible in Paris otherwise. The plants we collected came from the Alpine garden, that mimics a high-mountains climate, and greenhouses, that mimic desert, tropical forest (warm and cool) and New Caledonia climates. The considerations made by gardeners for replicating the microclimates of each plant we collected are outlined in Tables S1 and S2. They follow various protocols for the management of:

- **The soil quality.** The soil controls the pH, available nutrients, storage of heat and drainage of water, which has consequences on root temperature through the day and resources availability for the plant.[3] Different basic soil compositions are used and locally modified to adapt to the plants needs. In Tables S1 and S2, we make explicit the following compositions:
  - A1: Pozzolana, sieved topsoil, vegetable compost;
  - A2: Topsoil, limestone, rocks.
- **The watering frequency.** There are different watering protocols for the plants. The gardeners apply them at the level of individual plants. In Tables S1 and S2, we named the following protocols:
  - W1: Natural watering, or artificial watering every other day for 30 min when humidity is low;
  - W2: Watered once a day when the plant is in vegetative state, and three times a week when the plant is at rest (usually 6 months per year, over winter);
  - W3: Spring to Fall: heavy watering over short periods interrupted by dry periods. Fall to Spring: no watering. (adapted to arid plants).
- **The humidity.** The natural watering effect of morning dew is not reproducible in the Alpine garden; therefore the plant's conditions differ from their ideal climate. On the other hand, the humidity is precisely controlled in all the greenhouses.
- **The illumination.** The illumination of both the Alpine garden and the greenhouses comes from the Sun. It can be modified artificially over the seasons to protect the plants. For example, shade structures can be used in Summer to protect the plants from the light and heat in the middle of the day. The gardeners also play with the shadings and relative positioning to other plants and inside the greenhouse to expose the plants to the right amount of sunlight they need. Some of the greenhouses are whitewashed every Spring to minimize the sun transmission. The whitewash is gradually washed away by the rain over the year.
- **The winds.** The Alpine garden is protected for winds because it is installed in a cavity, 3.25 m below the level of the rest of the botanical garden.[3] In the greenhouses, the plants are also protected from wind.
- **The temperature.** The Alpine garden is exposed to Parisian seasonal temperatures, but some plants are covered in a hoop houses in winter for protection. On the other hand, the

temperature of the greenhouses is regulated. For each greenhouse, a minimum threshold is set for the nocturnal temperature (N) and diurnal temperature (D). When the temperature is too low, the heating level is increased on a daily basis. When the temperature is too high, the watering system is activated which increases the humidity and reduces the temperature.

- **The climate extremes.** In the Alpine garden, the protective effect of snow layers in the high mountains is artificially reproduced by covering some species with a plastic and wooden plate in winter, that limits frost. The greenhouses also allow reproducing climate extremes, such as high temperatures for the arid greenhouse, which can reach up to 45°C.

Species	shading	artificial control	watering	humidity	soil	temperature	microclimate
Dryas octopetala	full Sun	NA	W1	deficit	A1, no rocks	seasonal	alpine
Gentiana acaulis	full Sun	NA	W1	deficit	A1 + baltic blond peat	seasonal	alpine
Eremogone gypsophiloides	full Sun	NA	W1	deficit	A1	seasonal	alpine
Graptopetalum paraguayense	half Sun	hoop house in winter	once a week	no impact	A2 + sand	seasonal	alpine (desert mountains)
Minuartia stellata	full Sun	shade in summer plate structure in winter	W1	deficit	A1 + vegetable compost	seasonal	alpine
Opuntia microdasys subsp. Rufida	full Sun	hoop house in winter	once a week	no impact	A1 + sand	seasonal	alpine (desert mountains)
Ramonda myconi	full shadow	plate structure in winter	W1	deficit	A1 + sand + vegetable compost	seasonal	alpine
Saxifraga hostii	full Sun	NA	W1	deficit	A1 + vegetable compost	seasonal	alpine
Tulipa didieri	full Sun	NA	W1	no impact	A1 + vegetable compost + gravels	seasonal	alpine
Marchantia polymorpha	full shadow	NA	natural	no impact	milestone	seasonal	worldwide
Lemna sp.	full Sun to half Sun	NA	natural	no impact	water	seasonal	worldwide

Table S1: Growth conditions of plants collected in the Alpine garden, collection of the botanical garden of the Museum National d'Histoire Naturelle.

Species	shading	artificial control	watering	humidity	soil	temperature[1]	microclimate
Sinningia leucotricha	half shadow	glass whitewashed in spring, extra glass window in summer	W2	>80%	topsoil, compost, filtering medium	N: 16°C D: 20°C	tropical warm
Selaginella kraussiana	full shadow	glass whitewashed in spring	kept humid	>80%	vegetable compost	N: 16°C D: 20°C	tropical cool
Dictymia mettenii	full shadow	glass whitewashed in spring, blinds in summer	kept humid	>80%	pine bark, coco fiber	N: 16°C D: 20°C	tropical cool
Columnea guttata	half shadow	glass whitewashed in spring, blinds in summer	once a day	>80%	topsoil, compost, filtering medium	N: 16°C D: 20°C	tropical warm
Dischidia ruscifolia	full Sun	glass whitewashed in spring	3 times a week	70%	50% mineral (sand, pumice) 50% vegetable compost	N: 16°C D: 20°C	tropical warm
Pittosporum cherrieri	full Sun	NA	kept humid	70-85%	ericaceous compost (pH 5)	N: 14°C D: 22°C	forest New Caledonia
Bocquillonia castaneifolia	half Sun	NA	kept humid	70-85%	ericaceous compost (pH 5)	N: 14°C D: 22°C	mining maquis
Hereroa calycina	full Sun	NA	W3	60-70%	mineral (sand, pumice)	N: 10°C D: 13°C	desert
Adenium obesum v multiflorum	full Sun	NA	W3	60-70%	mineral (sand, pumice)	N: 10°C D: 13°C	desert
Monophyllaea horsfieldii[2]	half Sun	Kept under a hood	kept humid	>95%	spontaneous	28°C	tropical warm
Begonia platanifolia	half shadow	glass whitewashed in spring	once a day	75-100%	60% topsoil 40% mineral	N: 18°C D: 22°C	warm undergrowth

Table S2: Growth conditions of plants collected in the greenhouses, collection of the botanical garden of the Museum National d'Histoire Naturelle. [1] The temperatures indicated correspond to minimal nocturnal (N) and diurnal (D) temperatures. See text for more details. [2] Grown under neon light

References

[1] A. Lahlou, H. S. Tehrani, I. Coghill, Y. Shpinov, M. Mandal, M.-A. Plamont, I. Aujard, Y. Niu, L. Nedbal, D. Lazár, et al., *Nature Methods* **2023**, *20*, 12 1930.

[2] R. Chouket, A. Pellissier-Tanon, A. Lahlou, R. Zhang, D. Kim, M.-A. Plamont, M. Zhang, X. Zhang, P. Xu, N. Desprat, D. Bourgeois, A. Espagne, A. Lemarchand, T. L. Saux, L. Jullien, *Nature communications* **2022**, *13*, 1 1.

[3] M. d'Histoire Naturelle (Paris), *Annales du Muséum d'Histoire Naturelle*, volume 3, **1805**.
